# As Simple As Possible, but Not Simpler: Exploring the Fidelity of Coarse-Grained Protein Models for Simulated Force Spectroscopy

**DOI:** 10.1371/journal.pcbi.1005211

**Published:** 2016-11-29

**Authors:** Mona Habibi, Jörg Rottler, Steven S. Plotkin

**Affiliations:** 1 Department of Physics & Astronomy, University of British Columbia, Vancouver, British Columbia, Canada; 2 Genome Sciences and Technology Program, University of British Columbia, Vancouver, British Columbia, Canada; University of Virginia, UNITED STATES

## Abstract

Mechanical unfolding of a single domain of loop-truncated superoxide dismutase protein has been simulated via force spectroscopy techniques with both all-atom (AA) models and several coarse-grained models having different levels of resolution: A Gō model containing all heavy atoms in the protein (HA-Gō), the associative memory, water mediated, structure and energy model (AWSEM) which has 3 interaction sites per amino acid, and a Gō model containing only one interaction site per amino acid at the C_*α*_ position (C_*α*_-Gō). To systematically compare results across models, the scales of time, energy, and force had to be suitably renormalized in each model. Surprisingly, the HA-Gō model gives the softest protein, exhibiting much smaller force peaks than all other models after the above renormalization. Clustering to render a structural taxonomy as the protein unfolds showed that the AA, HA-Gō, and C_*α*_-Gō models exhibit a single pathway for early unfolding, which eventually bifurcates repeatedly to multiple branches only after the protein is about half-unfolded. The AWSEM model shows a single dominant unfolding pathway over the whole range of unfolding, in contrast to all other models. TM alignment, clustering analysis, and native contact maps show that the AWSEM pathway has however the most structural similarity to the AA model at high nativeness, but the least structural similarity to the AA model at low nativeness. In comparison to the AA model, the sequence of native contact breakage is best predicted by the HA-Gō model. All models consistently predict a similar unfolding mechanism for early force-induced unfolding events, but diverge in their predictions for late stage unfolding events when the protein is more significantly disordered.

## Author Summary

Although experimentalists can now unfold single proteins in the lab by pulling them apart and measuring the force and extension, a clear idea of how the protein changes shape and loses structure during this process is currently missing. Molecular dynamics simulations can offer insight as to what is actually happening structurally when you pull a protein apart. However, typical simulations of processes that happen nearly instantaneously in the lab take weeks to perform, when every atom must be accounted for. Researchers have thus resorted to much faster “coarse-grained models”, where the system is simplified by removing select atoms and the remaining interactions rescaled, but the accuracy of such simulations are known to suffer as a result. How accurate or inaccurate are the current coarse-grained models in capturing the unfolding mechanisms of proteins? Our findings upon investigating this question suggest that, while coarse-grained models successfully capture early unfolding events of nearly-folded proteins, they suffer when trying to describe the late stages of unfolding in mostly-disordered proteins. By showing how coarse-grained models may fail to capture the accuracy of their more sophisticated but cumbersome counterparts, we can shed light on how to improve their reliability, increase their speed, and enhance their relevance in capturing biologically-relevant phenomena.

## Introduction

No other scientific discipline has been so challenged to match the standard of physics-based simplicity as molecular and cell biology, perhaps in parts due to the inherent complexity of the systems under study and to our incomplete knowledge of the structure and function of the living cell. In narrowing this gap, minimal models of proteins have been developed as a step towards the goal of finding an “irreducible element” that still captures at least some of the essential physics and can thus reproduce and predict experimental measurements [1, 2].

In this regard, minimal models have enjoyed success in testing, refining, and validating the conceptual foundations of the energy landscape theory of protein folding [3–7] as well as forced unfolding mechanisms [8]. A minimal model attempts to capture the essential dynamical behavior of a protein, while upholding the notion of simplicity along with its concommitant computational efficiency. In practice this involves coarse-grained (CG) representations of a protein with fewer degrees of freedom than the atomic level of description, simpler, phenomenological interaction potentials, and classical rather than quantum dynamics.

Various semi-quantitative comparisons between CG models and experiments have been made [9–11]. At present however, systematic tests comparing the accuracy of coarse-grained models with fully atomistic models are still in need. Fully-atomistic models of proteins have their own shortcomings, including the inability of current atomistic force-fields to fold some proteins such as ubiquitin, a problem which has however been addressed recently and at least partially resolved [12]. However, all-atom models have now been successful in folding small proteins [13, 14], elucidating the binding properties of small-molecule drugs [15], and characterizing complex molecular processes such as ribosomal translation [16].

Steered molecular dynamics (SMD) simulations can provide an *in silico* realization of experimental force microscopy studies [17–19], where a force can be applied to a single protein– by optical tweezers for example– to unfold it [9, 10, 20]. Such computational studies can reveal details of the conformations of proteins during forced unfolding at atomic resolution. Force-extension curves obtained from atomic force microscopy (AFM) or optical trap assays generally display a saw-tooth pattern, where each partial unfolding event corresponds to a sudden drop in resistive force [9, 10, 20–22].

Our objective in this paper is to evaluate several CG models in SMD simulations by comparing the unfolding mechanisms predicted by each model to those predicted by a reference all-atom simulation under the same conditions. To this end, we construct scaling procedures such that the time, energy, and force scales can be meaningfully compared, and we develop several different metrics that each provide a different viewpoint of the unfolding dynamics.

It has been shown that the dynamics of small, globular proteins is well-depicted by all-atom force fields with CHARMM22* with explicit TIP3P water molecules as solvent [14]. Atomistic simulations with explicit solvent, however, are limited in length and time scales of order 100nm and a few *μ*s, unless specialized hardware is used [23]. Simulating the complete unfolding process of a full protein in explicit solvent is currently unfeasible if one wishes to simulate the unfolding mechanism with the same pulling rates as in experiments, and obtain comparable statistics. Thus, to simulate and sample large systems, coarse-grained models are required, because the energy function can be evaluated rapidly and the resulting molecular dynamics does not require a short time step. Various aspects of the protein dynamics and folded structures are successfully captured by structure-based Gō-like models [1, 24–27], in which the protein is biased towards its native folded state by native interactions. An interesting question is whether structure-based models can accurately capture the dynamics and the intermediate conformations of partially unfolded proteins during the mechanical unfolding process [9, 10]. Here we consider three Gō-like models at different levels of resolution: the Associative memory, Water mediated, Structure and Energy Model (AWSEM-Gō) [27]; a heavy-atom Gō model [25] that considers all atoms except hydrogen; and a one bead per residue *C*_*α*_-based Gō model [24].

Several previous studies have compared CG models to all-atom simulations and experiments [9, 10]. Nevertheless, none of these studies have taken into account that effective time and energy scales must be normalized for meaningful comparison. There is some disagreement whether or not the unfolding pathways predicted by structure based models agree with all-atom simulations or experimental observations [9, 10]. The authors of ref. [10] propose that the unfolding pathway from both CG models of titin I27 domain protein and all-atom implicit solvent simulations are not consistent with the experimental results even at low pulling speeds. On the other hand, CG pulling simulations of T4 lysozyme in Ref. [9] qualitatively agree with the experimental findings [28–30]. Sun *et al.* [31] have compared structure-based Gō models and experiments using force-clamp simulations; these comparisons show general agreement but often fail when sequence details are important in determining the weights of folding intermediates.

In this paper, we study the forced unfolding process of a monomer of a loop-truncated variant of superoxide dismutase (SOD1). SOD1 was the first protein discovered in which mutations had an autosomal-dominant causal relationship to amyotrophic lateral sclerosis (ALS) [32, 33], an invariably fatal motor neuron degenerative disease characterized by progressive loss of motor neurons [34], with a lifetime risk by age 70 of about 1/1000 [35]. The loop-truncated variant of SOD1 has loops IV (residues 49–81) and VII (residues 124–139) replaced with short Gly-Ala-Gly tripeptide linkers; here we denote this variant simply as tSOD1 [36, 37]. tSOD1 consists of a *β*-barrel tertiary fold containing 8 *β*-strands and 110 residues. While full-length SOD1 readily forms a homodimer, tSOD1 is obligately monomeric. Moreover, the disulfide bond between C57 and C146 is no longer formed due to the truncation of loop IV and removal of the putative C57. In experimental protein constructs, the remaining cysteines are mutated (C6A/C111S/C146S) to avoid intermolecular crosslinking; we employ the same construct here. In what follows, we first present the details of each model and the simulation set-up. We next describe the normalization of time and energy across models, by calibrating the pulling-rate, temperature, and force in the CG models with respect to the all-atom model. Then we discuss the force-extension curves we obtained, the evolution of structure as the protein is unfolded, and the predictions of the unfolding pathways provided by each model. We finally conclude and briefly discuss the implications of our results.

## Methods

### Simulation models

The aim of this study is to simulate the pulling process of the loop truncated SOD1 protein [37], and compare the results of an all-atom model with several coarse-grained (CG) models. The experimental structure of the tSOD1 monomer can be found as chain A of PDB ID 4BCZ. Force spectroscopy simulations were carried out by tethering both termini with a harmonic potential. The last residue (C-terminus) is then moved along the vector from C- to N-terminus with constant velocity of 1 m/s. The stiffness of the spring that imparts the pulling force on the protein was set to 1000 kJ/(mol · nm^2^). Experimental pulling speeds in atomic force microscopy (AFM) vary widely between 10^−8^-10^−2^ m/s [38–40], while typical speeds in atomistic simulations are significantly faster, also varying widely between 1–1000 m/s [9, 10, 22, 41]. Simulating and sampling the unfolding mechanism of a full protein in explicit solvent with the same pulling rate as in experiments is currently not feasible. The faster pulling rates in simulations may preempt slow dynamical transitions on the unfolding pathway that would otherwise occur at slower rates. A systematic study of the dependence of the unfolding mechanism on pulling rate for the present system is an interesting topic for future research.

**Fig 1:**
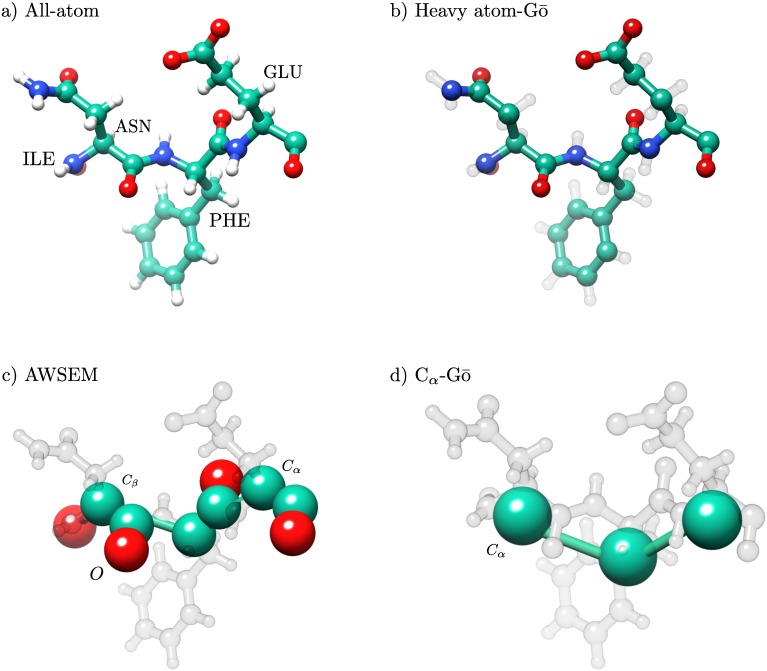
Representation of residues, Glu-Phe-Asn-Ile in AA, HA-Gō, AWSEM, and *C*_*α*_-Gō models. The resolution of the structure of the models decreases from a) to d). In the all-atom model, all hydrogen atoms are present (white beads). A protein structure in the HA-Gō model includes only heavy atoms. In the AWSEM model, there is no explicit representation of side chains. In the HA-Gō and *C*_*α*_-Gō models, the protein is biased towards its native state through attractive Lennard-Jones (LJ) interactions between residues that form a contact in the native state. The size of the beads in the picture is schematic only and does not represent the size of the atoms in the CG models. Schematics were constructed using Chimera [42].

Four different types of force fields and protein models were considered: an all-atom (AA) simulation in explicit solvent, a heavy atom Gō model (HA-Gō) [25], the Associative memory, Water mediated, Structure and Energy Model (AWSEM-Gō) [27], and a *C*_*α*_-Gō model [24] in order of decreasing resolution. In the HA-Gō model [25], all heavy atoms are present. The AWSEM-Gō [27] model is an associative memory Hamiltonian model with a three-bead representation per amino acid. In the *C*_*α*_-Gō [24] model, each amino acid is represented by only one bead [11, 24]. Note that in Gō models, only native interactions are attractive, while non-native interactions are purely repulsive. Further description of the Gō model including interaction potentials is given in the specific models sections below. Fig 1 shows a representation of four amino acids in each of the models. Pulling simulations were repeated 20 times for each model, with the same initial structure but different random seeds.

#### All-atom (AA) model

We used the CHARMM22* force field [43] to model a monomer of the loop-truncated SOD1 protein [37] with the TIP3P [44, 45] water model. All-atom simulations were carried out with the molecular dynamics code GROMACS-4.6 [46, 47]. To obtain the initial configuration for the pulling simulation, the PDB structure was energy minimized and equilibrated for 20 ns in an isobaric ensemble (NPT) simulation with a salt concentration of 0.15 M. The average size of the simulation box is 6.0 × 6.0 × 64.1 nm^3^ with 75,235 water molecules, 211 Na^+^ ions, and 208 Cl^-^ ions. A time step of 2 fs was used with the LINCS algorithm [48]. All all-atom simulations were performed in an isobaric ensemble (NPT) with a constant temperature T = 300 K and pressure p = 1 atm. The temperature of the protein and the solvent were kept constant with two separate thermostats [49–51]. The velocity rescaling algorithm with a stochastic term was used as thermostat for both protein and solvent [52]. The pressure was kept constant using the Parrinello-Rahman algorithm with a weak coupling of 1 ps [53]. Lennard-Jones interactions (LJ) were truncated at 1.4 nm, and the particle-mesh Ewald method [54] was used for the electrostatic interactions.

#### Heavy atom-Gō model (HA-Gō)

In the HA-Gō model [25], all heavy atoms (non-hydrogen) are present and the potential function is only defined by the native state. Any two heavy atoms that are within a cut-off distance 0.6 nm in the native state and are three or more residues apart are defined to form a native contact. In this system, the energy per contact for native interactions is *ϵ*_*c*_ = 0.43 *k*_*B*_*T*. The interactions between these non-bonded atom pairs are modeled by a 6–12 LJ potential [25, 55] and the separation corresponding to the potential energy minimum between pairs is set to the separation distance between pairs of atoms in the native PDB structure. Atoms that are not in contact in the native state are given a purely repulsive interaction given by [25] *U*(*r*) = ∑_*nn*_ *ϵ*(2.5Å/*r*)^12^, with uniform values of *ϵ*_*nn*_ = 0.01 *k*_*B*_*T*. Bonded atoms are modeled by harmonic bond and angle potentials, along with dihedral potentials [25]. The HA-Gō simulations were carried out with GROMACS-4.5 [46].

GROMACS input files were generated from the PDB structure using the SMOG [56] web server. The time step was set at 2 fs. The simulations were performed at constant temperature of 95 K (see below) using a Langevin thermostat with time constant of 1 ps. The initial configuration of the pulling simulations was obtained after 1 ns equilibration at the desired temperature.

#### AWSEM-Gō model (AWSEM)

The AWSEM-Gō (AWSEM) model is a coarse-grained protein force field [27] that is based on biophysical properties of the protein structure such as hydrogen bonding, water-mediated interactions, as well as a bioinformatic-based local structure biasing term. Each residue is represented by the position and relative orientation of its *C*_*α*_, *C*_*β*_ and *O* atoms in the backbone. The bioinformatic or “fragment-memory” term is

$$\begin{array}{c} V_{FM}=-\lambda \sum_{m}\sum_{ij}exp\left[-\frac{{\left(r_{ij}-r_{ij}^{m}\right)}^{2}}{2\sigma_{ij}^{2}}\right] \end{array}$$ (1)

where the outer sum is over aligned memory fragments, and the inner sum is over all possible pairs of *C*_*α*_, *C*_*β*_ atoms within the memory fragment that are separated by two or more residues [27]. *r*_*ij*_ denotes the instantaneous distance between the atoms, $r_{ij}^{m}$ is the corresponding distance in the memory fragment, *λ* is a scaling factor that can be used to change the strength of *V*_*FM*_, and *σ*_*IJ*_ =(1Å) |*I* − *J*|^0.15^ is a sequence separation-dependent width.

Note that *V*_*FM*_ is nonlocal, involving spatially-separated atomic pairs. For this study, we only used the available experimental information for the truncated SOD1 protein in the PDB in the database of memories, making the memory component of the model an effective Gō model. The total potential is [27]

$$\begin{array}{c} V_{total}=V_{FM}+V_{backbone}+V_{contact}+V_{burial}+V_{helical}. \end{array}$$ (2)

*V*_*backbone*_ maintains the protein backbone geometry through chain connectivity, bond, angle, dihedral angle, and excluded volume interactions, using backbone reconstrution assuming an ideal peptide bond. *V*_*contact*_ is an amino acid-dependent tertiary interaction term, consisting of a pairwise additive direct term, along with a many-body water mediated term.

The *V*_*burial*_ term represents the preference of an amino acid of a specific type to be buried inside the protein or to be on the surface, and *V*_*helical*_ is an explicit hydrogen bonding term that acts between the carbonyl oxygen of residue i and the amide hydrogen of residue i + 4, reconstructed from the coarse-grained model assuming an ideal peptide bond. A detailed description of the structural model and the force field can be found in Ref. [27].

For this model, the initial conformation was equilibrated for 1 ns before pulling. The AWSEM simulations were performed with the LAMMPS simulation package [57]. A time step of 5 fs and a Langevin thermostat with a time constant of 1 ps was used to keep the temperature constant at T = 319 K (see below for determination of the simulation temperatures).

#### *C*_*α*_-Gō model

The simplest model that we studied is the *C*_*α*_-Gō model, in which each amino acid is represented by one bead centered on their *C*_*α*_-atom positions [24]. This bead-spring protein model is biased toward the native state by an attractive 10–12 Lennard-Jones potential, set only between residues that are in contact in the native structure, as determined by a cutoff distance of 0.6 nm between any pair of heavy atoms. Pairs of residues may have one or more contacts depending on how many heavy-atom pairs are within the cutoff distance in the native states, thus the net interaction energy between residues is generally heterogeneous. The separation at the minimum of the potential for each pairwise interaction is set to the corresponding separation between the *C*_*α*_-atoms in the native PDB structure. The geometry of the backbone in the native state is modeled by harmonic potentials for angles and four-body dihedral potentials. For residues that are not in contact in the native state, the excluded volume diameter of each CG residue is ∼0.4nm [55]. The *C*_*α*_-Gō representation is a popular CG model and has been used extensively in studies of protein folding/unfolding mechanisms [1, 2, 9–11, 24].

The initial configuration of the pulling simulation is obtained after 1 ns equilibration. All parameters for the *C*_*α*_-Gō model were obtained from SMOG default values [56]. The time step for the *C*_*α*_-Gō model is set to 0.004 LJ time units. A Langevin thermostat with time constant of 12 LJ time units was used to keep the temperature constant at T = 142 K.

### Native and non-native contacts

To compare the mechanical unfolding pathway of the protein in the all-atom and coarse-grained models, we computed the number of native contacts of all configurations during the pulling simulations. The definition of a native contact is the same throughout this paper. We calculated the native contacts for pairwise distances of all the moieties *i* and *j* in each model for any protein structure (these may be heavy atoms, or coarse-grained residues). The fraction of the native contacts *Q* for conformation *X*, *Q*(*X*), is defined as

$$\begin{array}{c} Q\left(X\right)=\frac{1}{\left|S\right|}\sum_{(i,j)\in S}\frac{1}{1+\exp[\beta^{0}\left(r_{ij}\left(X\right)-\lambda r_{ij}^{0}\right)]}, \end{array}$$ (3)

where *r*_*ij*_(*X*) is the distance between moieties *i* and *j* in conformation *X*, $r_{ij}^{0}$ is the distance between the corresponding moieties *i* to *j* in the native state conformation, *S* is the set of all pairs of native contacts (*i*, *j*) belonging to the native structure. Amino acids having a native contact must be separated by four or more residues in the primary sequence and $r_{ij}^{0}\;<\;r_{cut}$ (*r*_*cut*_ is a model-dependent cutoff distance given in Table 1) in the native state [58], *β*^0^ is a smoothing parameter and the factor *λ* takes into account the fluctuations of the contacts.

**Table 1:** Parameters used to define contacts for each model (see Eq (3)). *β*^0^ is the smoothing parameter, *λ* takes into account the fluctuations of the contacts., and *r*_*cut*_ is the model-dependent cut-off distance.

Model	*β*^0^ [*nm*^−1^]	λ	r_cut_ [*nm*]
AA/HA-Gō	50	1.8	0.48
AWSEM	50	1.2	0.6
*C*_*α*_-Gō	50	1.2	1.2

As a result of adjusting *r*_*cut*_, different models exhibit approximately the same native contact map, and a scatter plot of the number of native contacts present during the pulling trajectory for the AA model vs the CG model exhibits a slope of unity (*y* ≈ *x*), (see Supporting Information S1 Fig). Table 1 summarizes the values of *β*^0^, *λ*, and *r*_*cut*_ for each model. For the all-atom and HA-Gō models, the same set of parameters were used as the models share the same structure. The number of contacts for pairs of residues in the *C*_*α*_-Gō model were weighted with respect to the number of contacts between the same pair in the native state of the protein in the all-atom model, i.e. a given pair of residues could have more than one contact between them, in proportion to how many of their heavy atoms were in contact.

All new contacts that are formed during the simulations between moieties *i* and *j* are considered non-native contacts if the distance between *i*, *j* in the PDB structure is larger than *r*_*cut*_, see Table 1 for values of *r*_*cut*_ in each model. To count the total number of non-native contacts in configuration *X*, we introduce a smooth function that interpolates between 1 and 0 as distance between *i* and *j* is increased, with a characteristic length scale *R*_0_ given by the mean of the distances between native pairs in the PDB structure: $R_{0}\;=\;\langle r_{ij}^{0}\rangle$. The smoothing parameter *β*_0_ and the factor *λ* are the same as for native contacts (see Table 1). The number of non-native contacts in configuration *X* is then:

$$\begin{array}{c} N_{nn}\left(X\right)={\sum_{\left(i,j\right)}}^{\prime }\frac{1}{1+\exp[\beta^{0}\left(r_{ij}\left(X\right)-\lambda R_{0}\right)]}, \end{array}$$ (4)

*R*_0_ = 0.24, 0.46, 0.91 nm for the AA & HA-Gō, AWSEM, and *C*_*α*_-Gō model, respectively.

We use *Q* in our analysis of all models as a convenient order parameter on which to project the unfolding mechanism, independent of its accuracy as a kinetic reaction coordinate. In what follows, we will also look at other quantities describing unfolding, such as *β* sheet dissociation, and structural alignment of remaining parts of the native fold.

### Time and energy scales in CG models

The interpretation of “time” and “energy” in a CG model must be carefully considered. The energy landscape of CG models is generally smoother, due to softer interaction potentials, reduced degrees of freedom, and lack of explicit solvent molecules. A smoother potential energy surface leads to faster dynamics in comparison to all-atom forcefields. Therefore, the meaning of time in CG models is not the same as in all-atom explicit simulations. When comparing time, velocity, energy, and forces in CG models and all-atom force fields, we should interpret the results with respect to an “effective” energy and time in the system.

#### Normalizing temperature scales

**Fig 2:**
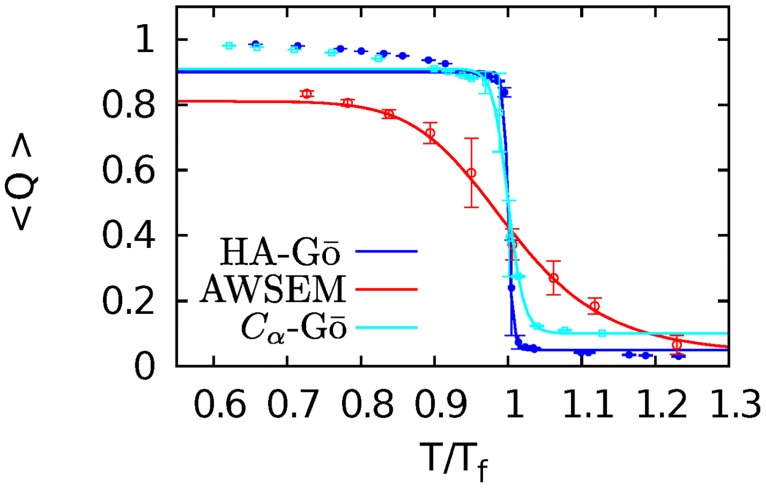
Melting curves for CG models. HA-Gō (blue), AWSEM (red), and *C*_*α*_-Gō (cyan). *T*_*f*_ = 106, 358, 158K for HA-Gō, AWSEM, and *C*_*α*_-Gō respectively. The fraction of native contacts is plotted as a function of temperature, normalized to the respective folding temperature for each model. Data bracketing the transition region is fit to Eq 5 to yield the solid lines. Error bars are for the correlated MD trajectories in the AWSEM model are obtained by the renormalization group method of Flyvbjerg et al [59]; the other models plotted here have data obtained from REMD methods, and thus use the standard error of the mean.

To be able to compare the CG and all-atom simulations at the same effective temperature, we performed all simulations at 90% of the folding temperature *T*_*f*_ of the protein in each model. Fig 2 shows the thermal melting curves for each of the CG models as a function of *T*/*T*_*f*_. To obtain the melting temperatures of the CG models, we ran replica-exchange molecular dynamics (REMD) simulations on the HA-Gō and *C*_*α*_-Gō models. To calculate 〈*Q*(*T*)〉 for the AWSEM model, we ran 50 direct MD simulations at each temperature *T*. Error bars for the AWSEM model are estimated from the correlated trajectories of *Q* versus time at each temperature. In determining the standard error of the mean, we perform a renormalization group method using block averaging to account for the effects of correlations in the trajectories. Each of the 50 MD trajectories started from the native state, and was sampled more frequently than the correlation time of each trajectory. The correlation time *τ*_*i*_ is found for each trajectory, and the data from time 0 to *τ*_*i*_ is removed. The remaining snapshots of each trajectory are then concatenated to one long (broken) trajectory and, for this correlated trajectory, the mean is found, and the renormalization group procedure of Flyvbjerg and Petersen is followed to obtain the converged standard error of the mean [59]. Implementing the standard error of the mean without renormalization on this data set gives smaller error bars than those obtained by the procedure we followed– about 60% of the size shown in Fig 2.

Convergence checks of the melting curves and details of the simulations are given in the Supporting Information S2 Fig. The data were fitted to the function

$$\begin{array}{c} \left\langle Q\left(T\right)\right\rangle =q_{u}\left(\frac{1}{1+{\text{e}}^{\Delta G(T)/RT}}\right)+q_{f}\left(\frac{{\text{e}}^{\Delta G(T)/RT}}{1+{\text{e}}^{\Delta G(T)/RT}}\right) \end{array}$$ (5)

where *R* is the gas constant and Δ*G*(*T*) = Δ*H* − *T*Δ*S*. The parameters *q*_*f*_, *q*_*u*_, Δ*H*, and Δ*S* are provided in Table 2.

**Table 2:** Thermodynamic parameters for unfolding (see Eq (5)). *q*_*f*_, *q*_*u*_ are fraction of folded and unfolded contacts, respectively. Δ*H* represents the enthalpy change. Δ*S* is the change in the entropy, and *T*_*f*_ is the melting temperature.

	q_f_	ΔH [$\frac{\text{kJ}}{\text{mol}}$]	ΔS [$\frac{\text{kJ}}{\text{molK}}$]	q_u_	T_f_ [K]	T_sim_ [K]
HA-Gō	0.90 ± 0.01	285.50 ± 0.63	2.68 ± 0.01	0.05 ± 0.01	106	95
AWSEM	0.82 ± 0.02	45.45 ± 7.25	0.127 ± 0.02	0.036 ± 0.04	358	319
*C*_*α*_-Gō	0.91 ± 0.02	125.48 ± 7.16	0.80 ± 0.05	0.1 ± 0.03	158	142

The melting or folding temperature *T*_*f*_ is defined as the temperature where Δ*G*(*T*_*f*_) = 0. From this procedure we obtained *T*_*f*_ = 358, 106, 158K for AWSEM, HA-Gō, and *C*_*α*_-Gō models respectively. The above procedure, wherein the unfolding enthalpy and entropy are treated as constants, is a crude approximation calorimetrically, and may be extended to either constant, or temperature-dependent unfolding heat capacity [60]. The above CG models all have explicitly temperature-independent interactions however, so it would be inconsistent to include such temperature-dependence in the calorimetric analysis.

The melting curve of the AWSEM model is significantly broader than the other coarse-grained models suggesting less folding cooperativity. The relative width Δ*T*/*T*_*f*_ of the thermal unfolding curves (from 80% to 20% of the folded baseline) are specifically 21% for the AWSEM model, 1% for the HA-Gō model, and 4% for the *C*_*α*_ model. This is to be compared with Δ*T*/*T*_*f*_ ≈ 4% for full-length apo, disulfide-reduced SOD1 [61].

The melting temperature of the all-atom model is taken as the experimental value *T* = 335 K [61], since the computational effort for performing either direct MD or REMD simulations on such a large protein in explicit solvent is prohibitive. Comparisons between experimental and computational melting temperatures by the Shaw group [13] show large scatter and little correlation. For the all-*β* proteins that were investigated however (WW domain and protein G), the experimental and simulated melting temperatures are 371 K and 372 K respectively for WW domain, while for protein G, the experimental and simulated melting temperatures are 340 K [62] and 345 K [13] respectively. The question arises as to the sensitivity of the AA-model results for the unfolding-mechanism upon the temperature of the simulation. To address this issue, we have performed additional simulations at both 290 K and 310 K, and analyzed the results in the Supporting Information, see S3, S4 and S5 Figs. In summary, the unfolding mechanism shows only small variations in this temperature range.

#### Normalizing time scales

The rate of pulling in each model system depends on each system’s internal time scale. To normalize time scales across models, one can scale the time in the CG models with respect to the AA model if a characteristic relaxation time for each model is known. To scale the time and thus normalize the rate of pulling, we measured a relaxation time, after mechanically perturbing each system, from the decay of the correlation function for the fraction of native contacts. To this end, we pulled several pairs of residues apart by 15 Å in separate simulations in each model, then removed the force and allowed the system to relax. The selected pairs are chosen randomly with two conditions: the residues in a pair should not be on the same *β*-strand, and a residue from each strand should be included in the list of 10 residues chosen. Residue pairs 13-69, 20-80, 35-45, 60-25 and 95-102 were pulled once to 15Å in all models, and then each perturbed system was allowed to relax without constraints. The normalized time autocorrelation of the fraction of native contacts $\langle \left(Q(t)-\bar{Q}\right)\left(Q(0)-\bar{Q}\right)\rangle$ for each model was calculated and fitted to a double exponential decay *A*_1_ exp(−*κ*_1_*t*) + *A*_2_ exp(−*κ*_2_*t*). The average values of *κ*_1_ and *κ*_2_ over all the perturbed system are given in Table 3, see Supporting Information S1 and S2 Tables for $\frac{A_{1}}{A_{2}}$, and $\frac{\kappa_{1}}{\kappa_{2}}$ for each perturbed residue pairs. To normalize pulling rates, we take the relevant time-scale in each model to be the inverse of the slower relaxation rate *t*_*CG*_ = 〈*κ*_1_〉^−1^ at *Q* in the folded state.

**Table 3:** Average relaxation rates obtained from fitting A_1_ exp(−*κ*_1_t) + A_2_ exp(−*κ*_2_t) to $\langle \left(Q(t)-\bar{Q}\right)\left(Q(0)-\bar{Q}\right)\rangle$.

Model	〈κ_1_〉 [ps^−1^]	〈κ_2_〉 [ps^−1^]
AA	0.0064	0.8661
HA-Gō	0.0910	16.4307
AWSEM	0.0147	0.4604
*C*_*α*_-Gō	0.2352	8.1569

In principle, the relaxation timescales could vary depending on the degree of unfolding. We have performed the same relaxation time calculations at an additional three values of the unfolding order parameter (Q = 0.7, 0.5, 0.3) and found that the relaxation rates vary by at most about a factor of two, and tend to decrease with unfolding for the coarse-grained models (see Supporting Information S6 Fig). No clear trend is apparent for the all atom model. Interpreting this result and separating the issues of different residual protein regions for different models at a given degree of unfolding vs the normalization of timescales across different models is interesting, but not straightforward. Moreover, the weak dependence of relaxation times implies such corrections would be small. Since the interpretation of distance is the same in the AA and all CG models, the pulling velocity *v*_*CG*_ for each CG model can be obtained from

$$\begin{array}{c} v_{AA}t_{AA}=v_{CG}t_{CG}. \end{array}$$ (6)

In the above relation, *v*_*AA*_ is the pulling speed in the AA model, *t*_*AA*_ = 156 ps, and *t*_*CG*_ is the characteristic time scale for the CG models. A physical pulling speed of *v*_*AA*_ = 1 m/s was used in all simulations.

## Results/Discussion

### Force spectroscopy simulations

**Fig 3:**
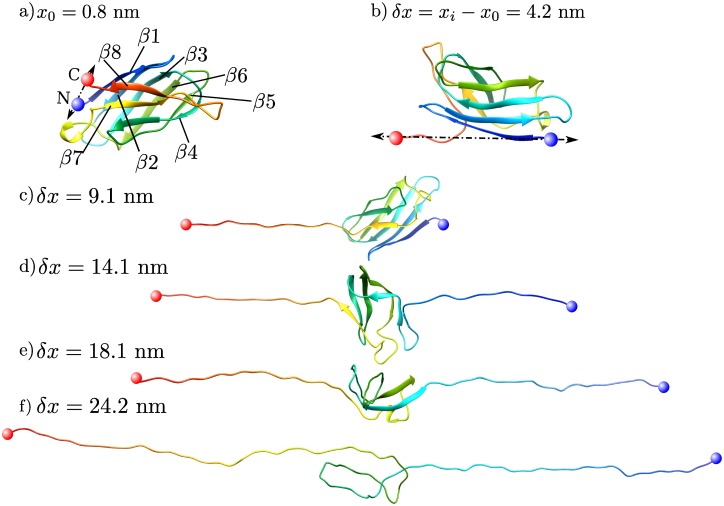
Snapshots of tSOD1 protein (PDB 4BCZ) in all-atom pulling simulations during the unfolding process. The tether points at the N-C termini are shown in blue and red spheres respectively. Panel a) shows the initial configuration of the protein and panels b-f show the protein at different extensions. The colors reflect the residues’ positions (their index) and map to a rainbow color gradient where *β*_1_ and *β*_8_ are blue and red respectively. The reported distances *δx* = *x*_*i*_ − *x*_0_ are the change in separation between the tethers, where *x*_0_ is the initial distance between tether points.

Fig 3 depicts representative snapshots of a pulling simulation in the all-atom model. The N- and C-termini are shown in red and blue spheres, respectively, and the structure of the protein is color-coded based on the residue index in the primary sequence. The reported values are the change in separation distance *δx* = *x*_*i*_ − *x*_0_, where *x*_0_ and *x*_*i*_ are the initial and instantaneous separation distance between tether points respectively (see Fig 3b). As we strain the protein, destabilized contacts between residues break, and regions of secondary structure in the protein are disrupted and dissociate. *β*-strands lose their native contacts, and locally unfold. The residues in the dissociated regions are then free to form turns or coil structures. In the all-atom simulations, the dissociation of the C-terminus at *δx* = 4.2 nm is the first unfolding event, see Fig 3b. In the unfolding trajectory shown in Fig 3c, we observe the dissociation of part of the N-terminus (*β*1-strand) at *δx* = 9.2 nm. In the snapshot shown in Fig 3f, the *β*5 and *β*6 sheets unravel last. At *δx* = 30 nm, the protein loses all its native contacts and forms a coiled chain.

### Force-extension curves (FECs)

**Fig 4:**
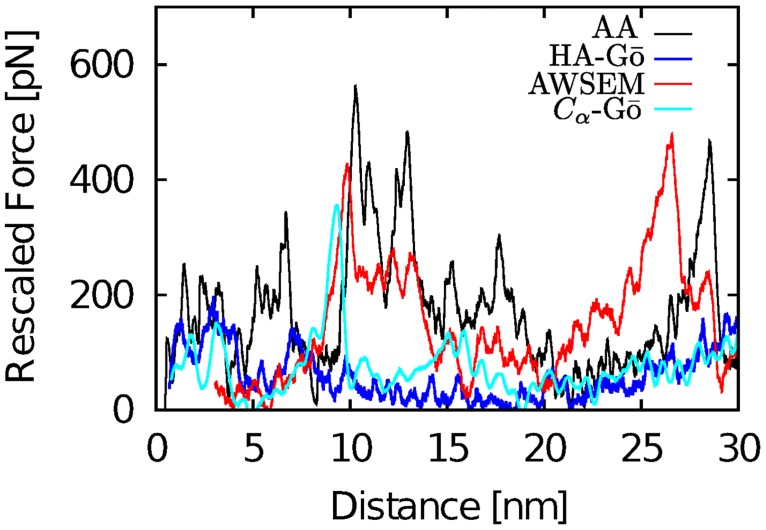
Force-extension curves for the unfolding of tSOD1 in all-atom (black), HA-Gō (blue), AWSEM (red), and *C*_*α*_Gō model (cyan). Because mechanical unfolding of a protein is a stochastic process, the unfolding force of a protein fluctuates randomly; the position of force peaks therefore varies between realizations. The force values for the CG models were rescaled with respect to free energy of unfolding of the protein *δx* = 30 nm as determined by the Jarzynski equality, see text.

In force spectroscopy simulations, the force ramps up until multiple contacts break, releasing the applied load. We observe multiple force drops (corresponding to multiple unfolding events) in the force extension curves. Fig 4 shows a force extension curve for one run of the AA (black line), AWSEM (red line), HA-Gō (blue line), and *C*_*α*_ model (cyan line).

#### Normalization of the unfolding force across models

In order to compare the force trajectories between AA and CG models meaningfully, we propose to normalize the forces in the coarse-grained models so that the total free energy change Δ*G*_*i*_/*k*_*B*_*T*_*i*_ upon unfolding, where *i* = HA, AWSEM, or *C*_*α*_, is the same as in the reference AA simulation. Since computing Δ*G*_*AA*_ is a challenging computational problem, we estimate an upper bound with the Jarzynski equality [63] directly from the nonequilibrium simulations. The force rescaling factor *α*_*i*_ for each model *i* is defined by applying the Jarzynski equality to the rescaled force *F*_*i*_:

$$\begin{array}{c} \ln\left\langle \text{exp}\left(-\beta_{i}\int_{0}^{L}\;F_{i}\left(x\right)\;dx\right)\right\rangle =-\beta_{AA}\Delta G_{AA} \end{array}$$ (7)

**Fig 5:**
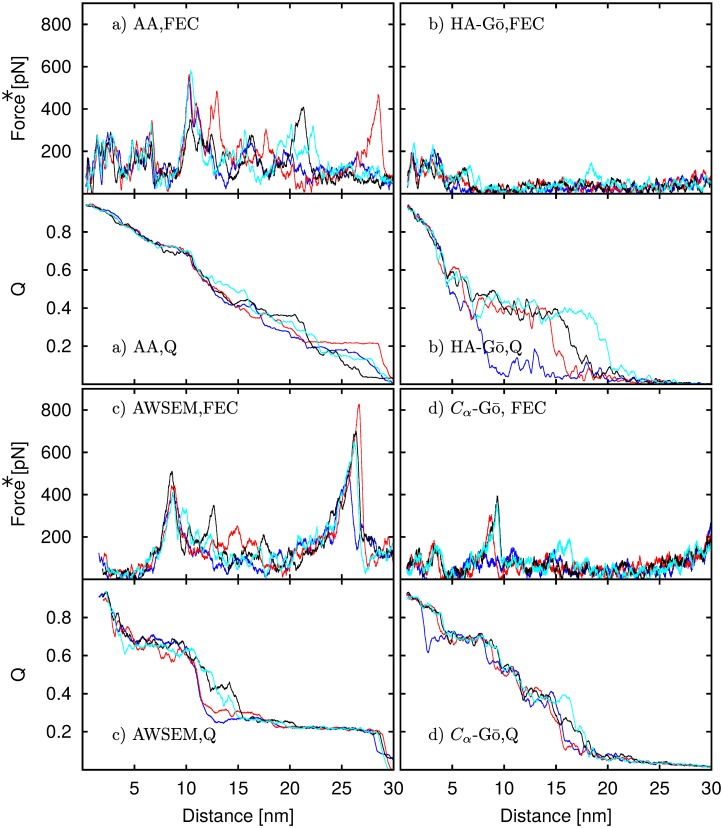
FECs and corresponding native *Q* curves for the a) All-atom, b) HA-Gō, c) AWSEM, and d) *C*_*α*_-Gō models for four runs. A high value of *Q* at small distances indicates that the protein is folded at the beginning of the pulling. As the protein is strained, *Q* decreases and finally approaches zero when the protein is fully extended. Each drop in the FEC corresponds to a drop in *Q*, indicating that the loss of native contacts releases the stress. Force* represent the rescaled force.

The rescaled force *F*_*i*_ reported below for model *i* exhibiting an unfolding force $F_{i}^{sim}$ is therefore $F_{i}\;=\;\alpha_{i}F_{i}^{sim}$; 〈〉 denotes an average over all 20 trajectories and *β*_*i*_ is the inverse temperature. We note that finite sample size corrections of the Jarzynski estimator for near-equilibrium perturbations have been discussed in the literature [64], but our limited data set does not permit us to ensure that these expressions are applicable. At *L* = 30nm the protein is fully unfolded but the worm-like chain tension is not significant, see Figs 4 and 5. This procedure yields *α*_*HA*_ = 4.72, *α*_AWSEM_ = 2.74, $\alpha_{C_{\alpha }}\;=\;8.27$, respectively. The above value obtained for Δ*G*_*AA*_ ≈ 860*k*_*B*_*T* is clearly an overestimate in part due to the large dissipation in the system and small sample size, however the relative values of forces between models may be unlikely to change significantly as sample size is increased: Convergence studies for *α* are given in the Supporting Information S7 Fig. Another important reason that Δ*G*_*AA*_ may be overestimated in the present assay relative to experimental values is that the unfolded protein is under substantial tension and consequently stretched. The free energy cost due to the consequent reduction in backbone conformational entropy simply due to restricted Ramachandran angles is of the order ∼200*k*_*B*_*T*.

#### Order parameter change during unfolding

For each model, we plot both the force-extension curves and the order parameter *Q* vs. extension for four different trajectories of the protein during mechanical unfolding in Fig 5. These trajectories were chosen to represent maximally different behavior in the set of simulations. For all models, as the protein unfolds, we observe a significant loss of native contacts, and finally Q approaches zero when the protein is fully extended. Each time the force ramps up, *Q* stays constant but then drops in lockstep with the drop in force. In general, each significant force drop corresponds to a decrease in the number of native contacts *Q*, which indicates the importance of the native contacts in unfolding events for all the models; both AA and AWSEM models have attractive interactions in addition to the native interactions.

The extension at which the protein loses most of its native contacts (*Q* < 0.2) is different for each model. For the AA model, *Q* ≈ 0.2 occurs when *δx* ≈ 25 nm, while for the HA-Gō, and *C*_*α*_-Gō models, the corresponding *δx* ≈ 20 nm, and for the AWSEM model, *Q* drops below 0.2 only after *δx* ≈ 27 nm. In one of the HA-Gō trajectories, the protein lost more than 80% of its native contacts at *δx* ≈ 10 nm. The AWSEM model features a significant drop of *Q* near the second force peak occurring at *δx* = 10–15 nm, which is absent in the other models. This drop is followed by a long plateau (15 nm < *δx* < 28) nm while the force ramps up. This behavior is in contrast to the HA-Gō model, where 3 out of 4 trajectories feature a large drop in Q towards the end of the unfolding trajectory (15 < *δx* < 20 nm). The AA and *C*_*α*_ models do not feature long plateaus and unfolding proceeds in smaller drops of Q.

In the AWSEM model, the contact potential for native contacts are only defined for *C*_*α*_ and *C*_*β*_ atoms in the backbone and not O atoms, see Eq 1. Consequently, the potential and obtained forces for the structure are calculated based on this definition (other terms in the model such as helical propensity and burial do include the oxygen atoms). However, in calculating *Q* for the AWSEM model, we employ the same definition as for all other models, i.e. we include all the heavy atoms within a cut-off distance. Thus there are technically extra contacts counted in the AWSEM model that result in a shift between the force drops and contact loss in Fig 5 panel C. For the HA-Gō and *C*_*α*_-Gō models the contact map and the native interactions are calculated only for atoms within a cut-off distance in the native states and include all the heavy atoms.

### Contact maps

**Fig 6:**
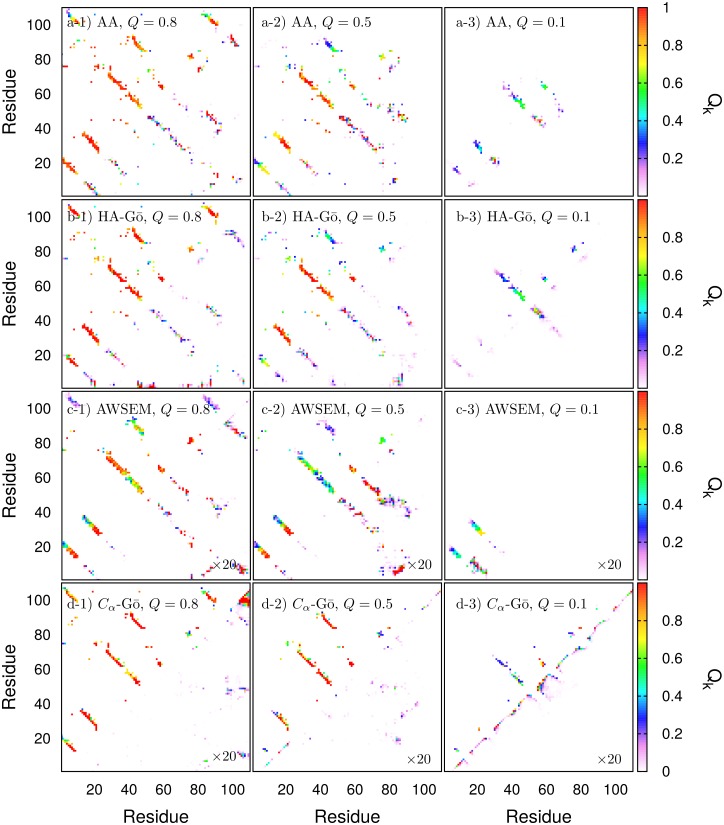
Contact-maps during forced unfolding. Top triangle: native contacts, bottom triangle: non-native contacts for *Q* = 0.8 (left column), *Q* = 0.5 (middle column), and *Q* = 0.1 (right column). Models are indicated in the legend of each panel. Non-native contacts are defined here as any contacts not present in the initial PDB structure. In the native contact-map, the color scheme is defined as red if all the native contacts between residues *k*, *l* from the PDB structure are present. Non-native contacts in the AWSEM and *C*_*α*_-Gō models are shown 20 times larger.

Contact maps of the protein averaged over the four runs in Fig 5 are depicted in Fig 6. In this work, native contacts are defined from the initial PDB structure. The upper triangle shows all native contacts at *Q* = 0.8, *Q* = 0.5, and *Q* = 0.1, respectively from left to right. The bottom triangle shows all non-native contacts, i.e. all new contacts that are formed during the course of the simulation. Since some of these residue pairs may also posess native contacts, they will appear in both maps. It is clear from the figure that native contacts induce the formation of many nearby contacts in the contact map when thermal fluctuations are taken into account. Native contacts between residues *k* and *l* are color coded by the thermal average number of contacts divided by the total number of contacts in the PDB structure, 〈*Q*_*kl*_(*Q*)〉. Non-native contacts do not have a particular reference structure to normalize with respect to. We thus color code the non-native contact between residues *k* and *l* by the frequency of occurrence of any non-native contacts between those residues in the ensemble of structures at *Q*, i.e. the fraction of conformations at *Q* that have at least one non-native contact between residues *k* and *l*. Here “at *Q*” means within the bin *Q* − *δQ*, *Q* + *δQ*, where *δQ* = 0.01.

At *Q* = 0.8, the native contact maps are approximately the same for all the models (see first column, *Q* = 0.8). As the protein unfolds from *Q* = 0.8 to *Q* = 0.5 (second column), the C-terminal domain unfolds completely in all the models. The contact maps predict the same general unfolding events until *Q* ≈ 0.5. As the protein unfolds further to *Q* = 0.1 (third column), the unfolding processes begin to take different pathways across models. For the HA-Gō and *C*_*α*_-models, the largest folded domain is located at residues 50–70, while for the AWSEM model the folded domain lies in residues 10–30. The remaining structured domain in the AA model is larger but only partially folded, consisting of residues 10–60.

We wish to emphasize that Fig 6 is not intended to illustrate the dominant unfolding mechanism for each model, but is simply an analysis of a subset of the unfolding trajectories, chosen only because they were distinct. A further analysis of the dominant unfolding mechanism will be discussed in the subsequent text and corresponding figures.

Fig 6 shows that, for all models having more than one interaction site per amino acid, non-native contacts consist largely of what one might call “near-native” contacts. For example in the AA and HA-Gō models, pairs of amino acids have several native contacts between their constituent atoms, however some atom pairs exist between these same amino acids that are not in contact in the native PDB structure. “Near-native” contacts would involve these particular atom pairs, and the non-native contact map, which does not have any native interactions by construction, appears quite similar to the native contact map as a result. The presence of native interactions increases the likelihood of proximal non-native interactions.

On the other hand, the *C*_*α*_-Gō model has only one interaction site per amino acid and so cannot exhibit near-native contacts. The non-native contact map is thus sparser than the other models, and involves distinct amino acid pairs. The short-range contacts reminiscent of *α*-helical structure that are observed at *Q* = 0.1 in the *C*_*α*_-Gō model are a consequence of the lenient cutoff used for contacts between *C*_*α*_ residues– the other models would show these non-native contacts as well, but because they have more degrees of freedom their cutoff distance for non-native interactions are shorter.

Non-native interactions between amino acid pairs wherein one amino acid has been shifted in primary sequence by one, i.e. from amino acids (*m*, *n*) to (*m* ± 1, *n*) or (*m*, *n* ± 1), can be induced by the shear forces between *β*-strands in the present assay, so that strands may slide over each other or reptate. Similar reptation has been observed in unbiased folding simulations of a *β*-hairpin [65]. Here, such “off-native” contacts are relatively common for all models that have more than one interaction site per amino acid; the relative numbers of amino acid pairs that partake in off-native contacts compared to the number of amino acid pairs partaking in native contacts, at the values of *Q* in Fig 6, are given in Table 4.

**Table 4:** Thermal averages of the number of off-native residue pairs/Number of native residue pairs.

Model	Q = 0.8	Q = 0.5	Q = 0.1
AA	216/256	174/204	64/98
HA-Gō	238/255	190/198	117/119
AWSEM	247/295	201/225	53/71
*C*_*α*_-Gō	24/200	30/132	43/64

### Residue contacts

**Fig 7:**
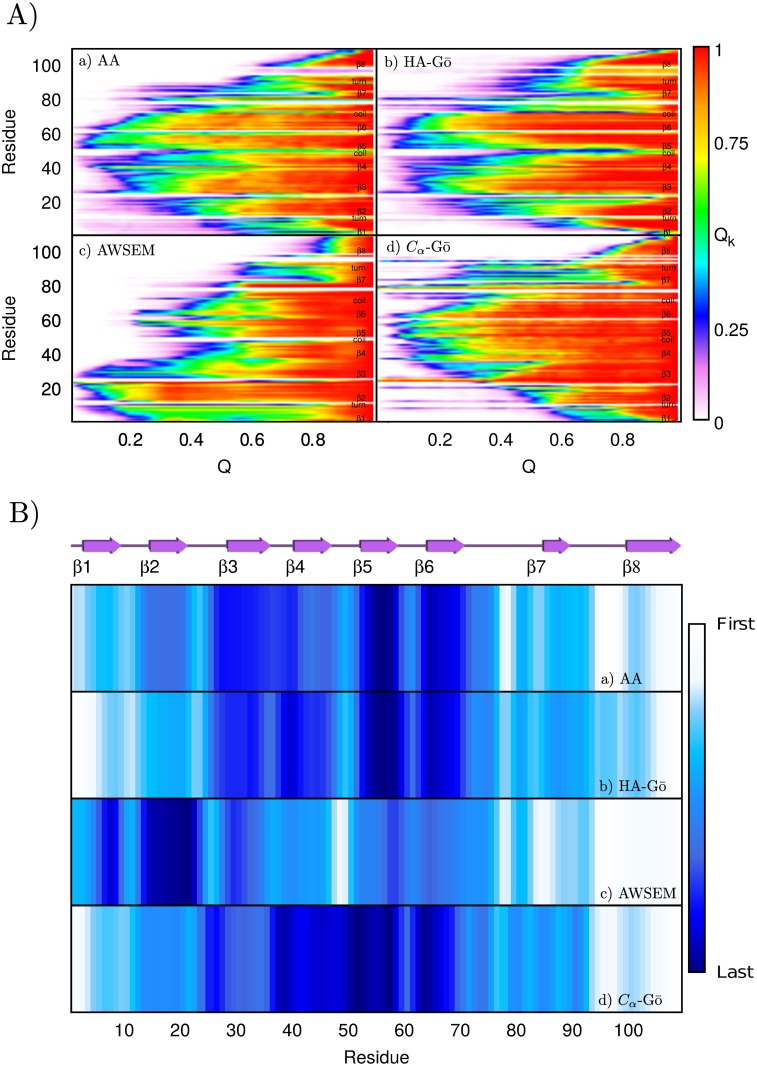
**A) Fraction of native contacts for each residue *Q*_*k*_(*Q*) vs. total number of native contacts (*Q*) a) All-atom, b) HA-Gō, c) AWSEM, and d) *C*_*α*_-Gō.** The color red shows the presence of all the native contacts and white represents a residue that shares no native contacts with the other residues in the protein. **B) Another representation of the loss of native structure as the protein is mechanically unfolded.** The color scheme represents which residue loses more than 50% of its contacts (*Q*_*k*_ < 0.5) first in *Q*. The white color shows the least stable residues and the dark blue represents the most persistent residues.

To determine the sequence of the unfolding residues, we monitored the number of native contacts for each residue during the pulling simulations. Fig 7A plots the average fraction *Q*_*k*_(*Q*) of a given residue *k* as a function of total Q for all models. To calculate *Q*_*k*_(*Q*), we normalize the number of contacts at *Q* by the number of contacts that residue *k* possesses in the native structure where *Q* = 1. Red color corresponds to *Q*_*k*_(*Q*) = 1 and white indicates *Q*_*k*_(*Q*) = 0, i.e. the residue has lost all its native contacts. The color scheme in Fig 7B represents the sequence (in terms of the global order parameter *Q*) by which residues lose more than 50% of their contacts during unfolding. The most persistent residues are colored dark blue, and the residues that are broken first in sequence are colored white. From Fig 7, it is clear that all models predict as first event the dissociation of the C-terminus, residues 100–110 (*β*8). Then, in the AA, HA-Gō, and *C*_*α*_-Gō model, the N-terminus detaches. The average unfolding pathways predicted by the AA model are very similar to the HA-Gō model, where residues in the N- and C-terminus dissociate first, and the contacts of residues 50–74 are broken last. In contrast, the sequence of unfolding in the AWSEM model starts from *β*8 and *β*7, and the last domains to rip off are *β*3 and *β*2.

In summary, the similarity between unfolding events depicted in Fig 7B may be quantified by computing the correlation coefficient between the degree of remaining structure for individual *β* strands (the similarity of the darkness of the bands for each model in Fig 7B). This gives the following correlation coefficients: between AA and HA-Gō: 0.94, between AA and *C*_*α*_-Gō: 0.86, and between AA and AWSEM: 0.62.

### Protein unfolding pathway

In order to determine whether there exists a well defined unfolding pathway of the tSOD1 protein, and if so, to compare it across models, we used the template modeling score (TM-score) [66] to compare the similarity between the protein structures of different pulling trajectories at the same Q. The TM-score for the alignment of two structures is defined as [66]:

$$\begin{array}{lll} \text{TM} & = & \frac{1}{L}\overset{N}{\sum }\frac{1}{1+{\left(\frac{d_{i}}{d}\right)}^{2}},\\ d & = & 1.24\sqrt[{3}]{\left(N-15\right)}-1.8, \end{array}$$ (8)

where *N* is the number of residue pairs, *d*_*i*_ is the distance between identical residues *i* in two structures, and *L* is the number of residues in the reference structure. The TM-score lies between 0 and 1; a TM-score of one indicates that the two protein structures are perfectly matched. Usually, two structures with TM-score higher than 0.5 are considered to have the same folded conformations, while uncorrelated protein structures have a TM-score of less than 0.2 [66]. Measuring the TM-alignment, as well as clustering of structures by TM-score, was performed by using Maxcluster (http://www.sbg.bio.ic.ac.uk/maxcluster) [67].

**Fig 8:**
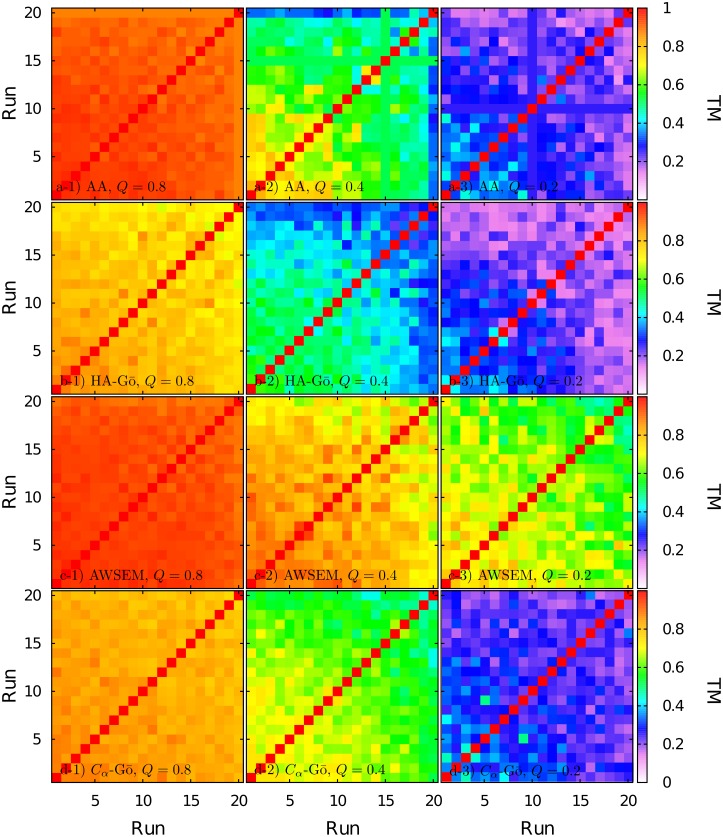
TM-score for folded segments of the protein structures for different runs at Q = 0.8, 0.4, 0.2 from left to right, for a) All-atom, b) HA-Gō, c)AWSEM and d) *C*_*α*_-Gō model. The color code runs from white (TM-score = 0) to red (TM-score = 1).

TM-scores of an all-against-all structure comparison of folded segment of protein structures obtained from each run for *Q* = 0.8, *Q* = 0.4, and *Q* = 0.2 are shown in Fig 8. The color code quantifies the TM-score of pairs of structures at the same value of *Q*, obtained from all pairs of trajectories: red color indicates perfectly matched structures, and white represents a TM-score of zero. For comparing the conformations, we only considered *C*_*α*_-atoms in the backbone for the *folded* region of the protein. This folded region at each *Q*-value was defined as a contiguous sequence of *n* residues with residue index *i* ≤ *j* ≤ *i* + *n*, where 〈*Q*_*i*_(*Q*)〉 > 0.5 and 〈*Q*_*i*+*n*_(*Q*)〉 > 0.5. The average here corresponds to the ensemble of states of all trajectories. If there is an unfolded region with more than 10 residues in between *i* and *i* + *n*, then the largest contiguous sequence of residues with 〈*Q*_*i*_(*Q*)〉 > 0.5 was considered.

In Fig 8, TM-scores for *Q* = 0.8 (see left column in Fig 8) are high for all four models, which indicates that at the beginning of the unfolding process, the backbone of the protein is very similar in the unfolding trajectories. The *C*_*α*_-model and the HA-Gō model exhibit slightly larger deviations between trajectories at this value of *Q*. As the protein unfolds further, at Q = 0.4 (second column in Fig 8), the TM-scores drop to lower values. In the AA model, the average TM-score of one trajectory (run 20) is 0.33, while other runs have higher TM-scores. For the HA-Gō model, values of the TM-score range between 0.3–0.6. In the *C*_*α*_-Gō model, the TM-scores range between 0.5–0.76. At the same *Q* = 0.4, the TM-scores in the AWSEM model are still much higher and vary between 0.6–0.94, which indicates the presence of one dominant pathway.

**Fig 9:**
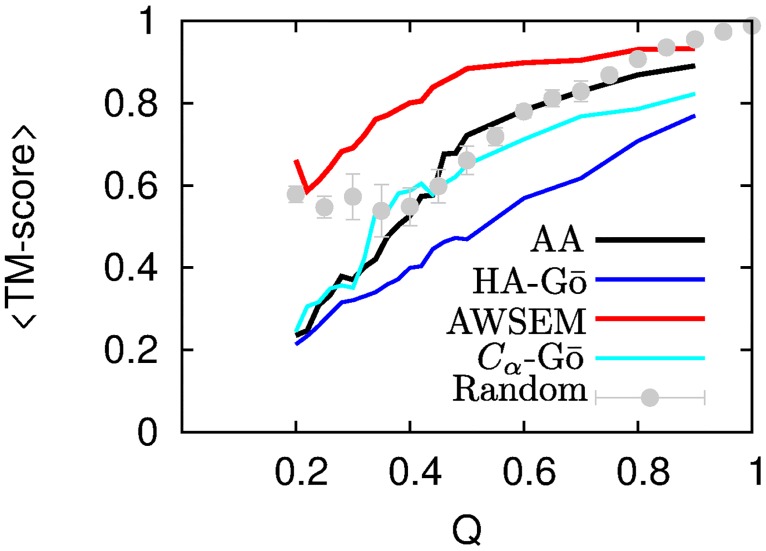
The mean TM-score between all trajectories as a function of *Q* for AA model (black line), HA-Gō (red dotted line), AWSEM (dashed blue), and *C*_*α*_-Gō (cyan line). The gray symbols show the average of TM-scores for a randomized set of unfolded structures (see text). The error bars correspond to the standard error in the mean.

It is clear from the large number of trajectories with high TM-scores that the AWSEM model exhibits a much stronger pathway behavior than the other models, which begin to balkanize into clusters of residual structure. This can also be clearly seen by plotting the mean TM-score between all *M*(*M* − 1)/2 trajectories (*M* = 20 here) as a function of *Q*, for all four models, see Fig 9.

At *Q* = 0.2, the TM-scores for AA, HA-Gō, and *C*_*α*_-Gō models have reached about 0.2, which is comparable to the TM-score of a random coil ensemble. This indicates a highly diverse residual structure between trajectories. The length of the residual folded structures at *Q* = 0.2 is only about 24, 38, 21, and 27 for AA, HA-Gō, AWSEM, and *C*_*α*_-Gō models. Thus, the AA, HA-Gō, and *C*_*α*_-Gō models predict multiple unfolding pathways for lower values of *Q*. On the other hand, the AWSEM model still has a fairly high TM-score; indicating that it predicts only one main unfolding pathway.

Two structures that are nearly folded at *Q* ≈ 1 are obliged to have a high TM score, while two structures at low *Q* are not so obliged. We thus also plot in Fig 9 a reference curve to compare the structural overlap. We construct this curve by taking a window containing a given number of residues (e.g. 50), and slide this window along all possible locations of the primary sequence (1–50, 2–51, etc.), to obtain a set of partial native structures, one structure for each window position. This process is repeated for all window sequence lengths. The native contacts Q are calculated for all of the structures, binned, and TM-aligned. This gives a randomized set of partially unfolded structures, which nevertheless lack thermal fluctuations and strain distortions, and so would tend to have larger TM-alignments when they overlap. Interestingly, this curve lies roughly between the AWSEM model and all other models, consistent with the strong pathway-like unfolding mechanism of the AWSEM model.

### Comparison across models

**Fig 10:**
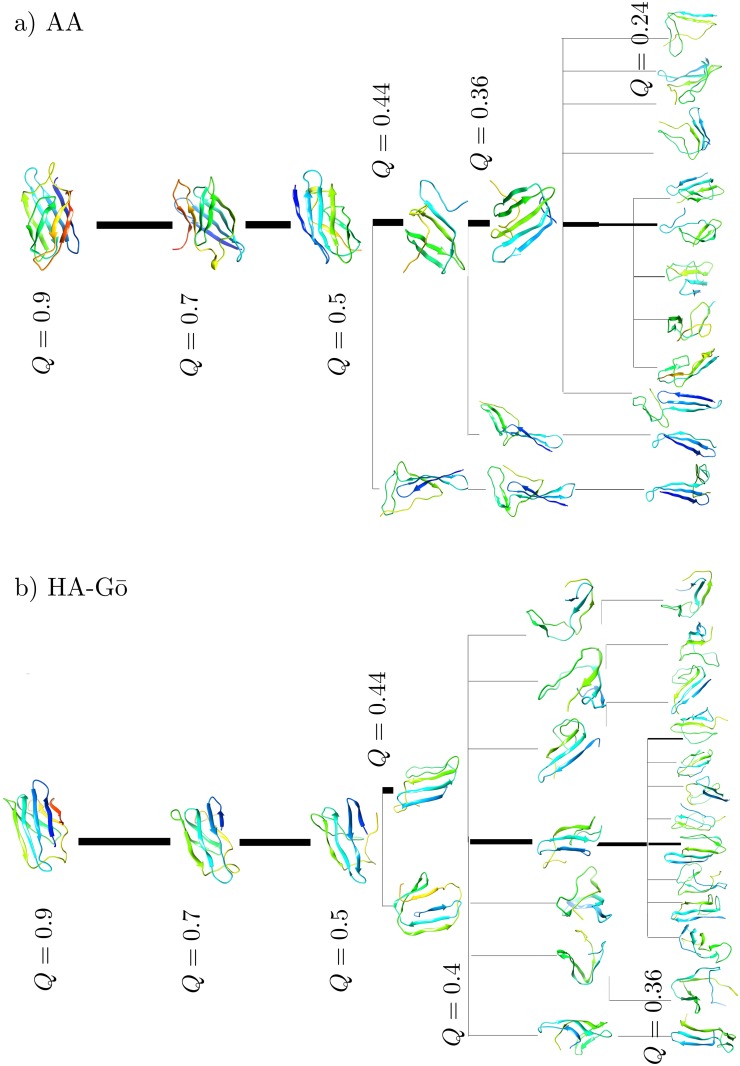
Cluster centroid conformations at different *Q* are shown for a) AA, and b) HA-Gō. The thickness of the each line is proportional to the fraction of total trajectories that connect the centroids of the clusters. For both models, there is a single pathway as long as *Q* > 0.44. As the protein unfolds more, the models predict multiple pathways. The dominant unfolding pathway corresponds to the thickest black line.

**Fig 11:**
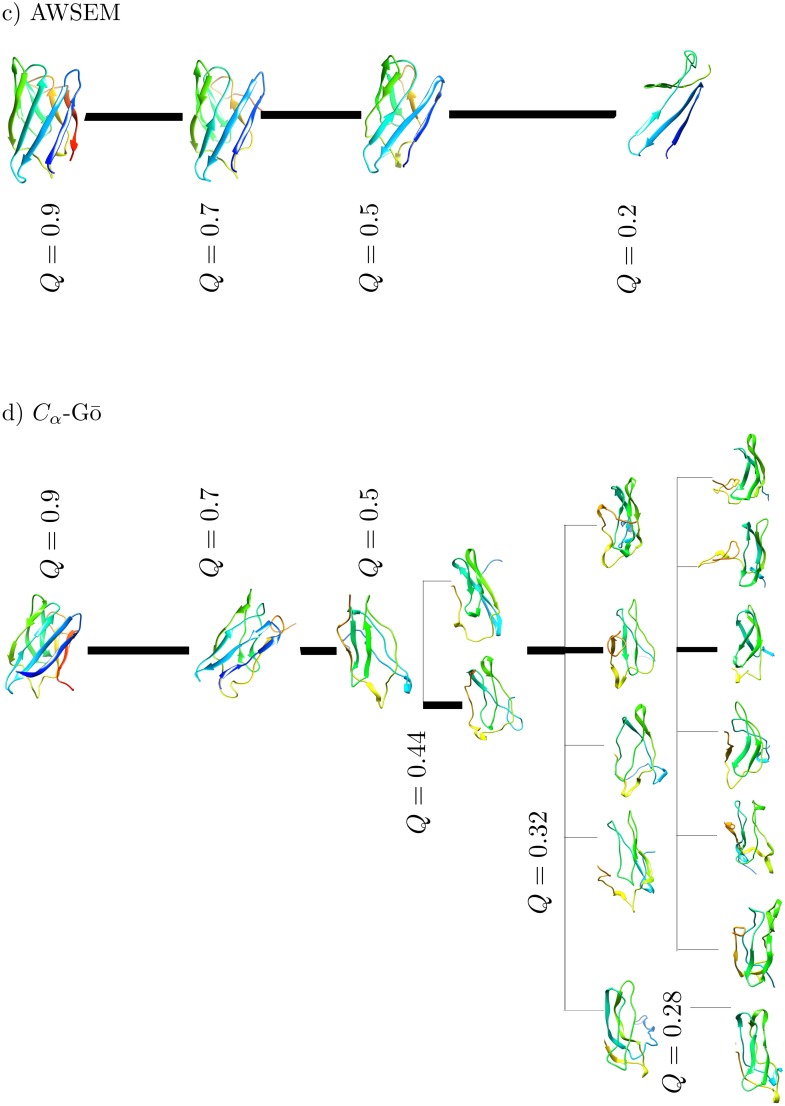
Cluster centroid conformations at different *Q* are shown for c) AWSEM, and d) *C*_*α*_-Gō model. The AWSEM model is characterized by only one unfolding pathway from *Q* = 0.9 − 0.24, in contrast to all-atom, HA-Gō and, *C*_*α*_-Gō models. The *C*_*α*_-Gō model has a single pathway of unfolding as long as *Q* > 0.44. The dominant pathway is shown with a thick black line. The thickness of the line is proportional to the fraction of total trajectories connecting the centroids in each cluster.

In order to more clearly render the unfolding pathways predicted by each model, we clustered the protein conformations based on the TM-scores during the unfolding at several different *Q*-values, see Figs 10 and 11. The structures shown are centroids of the corresponding clusters that emerge from the clustering analysis. A TM-score cut-off of 0.6 is used to define when configurations no longer belong to a given cluster. The coloring is based on the residue index, where the C-terminus of the structured protein is in red and the N-terminus is colored blue. The thickness of the lines is proportional to the fraction of total trajectories in each cluster.

As can be seen in Figs 10 and 11, each model predicts a dominant unfolding route, which is shown with a thick black line. All models predict one unique unfolding pathway until *Q* ≈ 0.44. Along this pathway, *β* strand 8 at the C-terminus loses structure first, however subsequent events differ between models. As the structure continues to unfold from *Q* ≈ 0.44 to *Q* ≈ 0.2, we observe multiple unfolding pathways in all models but the AWSEM model; see Fig 10 panel a) AA, b) HA-Gō, and Fig 11b) *C*_*α*_-Gō models. The protein structures from different pulling simulations in the above 3 models are distributed in multiple diverse conformations.

For the AA, HA, and *C*_*α*_-Gō models, *β* strand 1 on the N-terminus generally dissociates after *β* strand 8 at the C-terminus. In 3 out of 20 trajectories of the AA model however, *β* strands 1 and 2 were the last to unfold. This mechanism with *β* strands 1 and 2 unfolding last is the pathway observed in the AWSEM model. Generally, the last unfolding events involve breakage of contacts in *β* strands 5 and 6 in the AA model. The sequence of unfolding events along the main forced unfolding pathway in the AA model is *β* strand 8, then *β*1 and 7, *β*2, then *β*3 and 4, *β*6, and then finally *β*5. In the HA-Gō model, the sequence of unfolding of events is *β*8 and *β*1, then *β*2, *β*7, then *β*3 and 4, then *β*6 and finally *β*5 is the last domain to unfold, which is similar to the AA model. In the *C*_*α*_-Gō, the first unfolding event is also dissociation of C-terminal *β* strand 8, then *β*1, *β* strands 2 and 7, then *β*3, *β*4, *β*6, and finally *β*5.

In contrast to the above three models, the AWSEM model (Fig 11c) predicts only one unfolding pathway. In this pathway, the unfolding of the protein starts from the C-terminal *β* strand 8, then *β*7, *β*4, the C-terminal portion constituting roughly half of *β* strand 3, the N-terminal portion constituting roughly half of *β* strand 1, *β* strands 5 and 6 and the remainder of *β* strand 3, the remainder of *β*1, and *β*2. Strands 1 and 2 were the last to dissociate in all the 20 trajectories.

**Fig 12:**
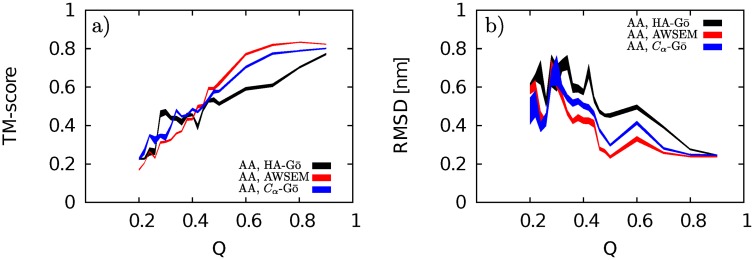
a) TM-score vs. Q, and b) RMSD vs. Q for AA & HA-Gō (black line), AA& AWSEM (red line), AA & *C*_*α*_-Gō (blue line). The thickness of each curve represents the statistical error obtained from the bootstrapping method. High values of TM-score (low values of RMSD) when the protein is partially unfolded (*Q* > 0.4) prove that all models predict a similar unfolding pathway until the protein is almost half folded. As the protein unfolds and more contacts break, each model predicts a different pathway. And finally, when there are about 20% contacts unbroken, there is no similarity between pathways.

We also compare the main pathway of unfolding of the AA-model with other models by calculating the TM-score between the AA model and the three CG models. For comparison across different models, TM-score was calculated using the program TM-align [68]. The conformations of the most populated cluster at *Q* in the AA model was compared to the corresponding conformations in the other models at the same value of *Q*. In order to compare CG with AA models, the TM-alignment only includes the *C*_*α*_ atoms in the backbone of the folded segment of the protein as described above. The TM-score versus *Q*, for pairs of two models, AA with HA-Gō (black line), AA with AWSEM (red line), and AA with *C*_*α*_-Gō (blue line), is depicted in Fig 12a.

A TM-score with a value of > 0.5 for a pair of proteins means that the structures are similar [68]. The observed high TM-scores between AA and all CG models for *Q* > 0.45 indicate that all CG models predict unfolding pathways similar to the AA model by this metric. Interestingly, in the range of *Q* between 0.45 and 1, the AWSEM model shows the best agreement with the AA model, and the HA-Gō model shows the least agreement.

As the protein is unfolded below *Q* ≤ 0.44, the TM-score shows a more sensitive dependence upon models. At *Q* less than about 0.25, the TM-scores have reached small values that would be expected for the alignment of random dissimilar structures.

We conclude therefore that all models predict similar unfolding pathways until the protein is about half unfolded, at which point the mechanisms begin to diverge from the AA model. The AWSEM model does not predict multiple pathways as the other models do, but the dominant pathway observed for the AWSEM model is structurally as similar to the AA model as any of the other CG models. None of the CG models can completely capture the unfolding mechanism at the lower values of *Q* for the AA model.

The above conclusion is recapitulated by analyzing the corresponding alignment between models using the more conventional metric of RMSD. Comparing the folded core of the AA model in the most populated cluster, as defined in Section “Residue Contacts”, to the same region in the CG models (most populated cluster, same sequence length as in the AA model) yields a plot of RMSD vs. *Q*, as shown in Fig 12b. By this metric, the AWSEM model again shows the best structural alignment (lowest RMSD) until *Q* ≈ 0.3, while the HA-Gō model shows the worst structural alignment.

### Conclusion

In this paper we explored the limits of validity of several structural-based coarse-grained (CG) models by comparing the unfolding mechanisms of a truncated variant of superoxide dismutase, when the protein is subjected to force-induced unfolding. An all-atom (AA), explicit-solvent model is used as the benchmark standard to which the other models are compared. A more desirable comparison would be with experimental data, however no experimental data exists for this particular system, and moreover the data that does exist for other systems does not have the atomic resolution that we have measured and compared with here. Unfortunately then such a comparison is not possible at present. One may entertain the possibility that one of the coarse-grained models could agree better with experiments than the all-atom model– at this time however, such comparisons are purely speculative and without any definitive precedent. To facilitate the present comparison between coarse-grained models and all-atom simulations, the models were normalized in terms of time, energy and force scales. We analyzed in detail several different metrics of the unfolding process: force-extension curves, evolution of contact maps, sequence of unfolding via loss of contacts involving a particular residue, and backbone alignment quantified by TM-score and RMSD.

We found that the force-induced unfolding mechanisms of all CG models differ to varying degrees from that in the AA model. Both HA and *C*_*α*_-Gō models do capture most aspects of the sequence of unfolding events. Comparing the all-atom model with a heavy-atom Gō model gives some clues as to the combined importance of both energetic heterogeneity of native contacts, and non-native interactions, in modulating the unfolding mechanism. The varying strength of native interactions can alter the free energy barriers to unfolding, possibly increasing them in special cases when polymer entropy cost is compensated by stronger interactions, but generally decreasing the folding/unfolding barrier [69–75]. The HA-Gō model does capture some effects of energetic heterogeneity by counting multiple contacts between amino acids involving large side-chains, but otherwise is an uncontrolled approximation that may return erroneous conclusions, particularly when electrostatic effects and solvation are important [76]. The HA-Gō model also captures entropic heterogeneity due to the variable backbone polymer length between residues participating in native contacts [77]. Unless they are strong enough to result in long-lived off-pathway intermediates, non-native interactions also generally decrease folding/unfolding barriers, and they can modulate unfolding mechanisms [70, 71, 78–80], or modify the diffusion coefficient along the folding reaction coordinate [81–86].

The HA-Gō model was the softest model examined, after suitable normalization was performed to equate the unfolding free energy across models. This is not obvious, given that it was not the most coarse-grained model that we had investigated. The *C*_*α*_ model closely follows as the next softest model.

The AWSEM model differed from all other models insofar as all folding trajectories follow a single unfolding pathway that does not branch out in the final stages, as one approaches the unfolded state. This pathway is part of the ensemble of paths observed in the AA model, however it is not the dominant pathway. On the other hand, the backbone structure predicted by AWSEM agrees best with the AA model while the protein is still mostly folded.

These findings substantiate that a combination of metrics is required to obtain a full picture of the unfolding dynamics. No single coarse-grained model studied here agreed best with all of those metrics simultaneously. It is perhaps surprising that the *C*_*α*_-Gō model, as the simplest model, does not perform substantially worse than the more detailed models. This finding may not be generically true however: A force peak specifically due to non-native interactions was observed in AA forced-unfolding simulations of DDFLN4, a predominantly *β*-sheet protein [87], which recapitulates experimental observations [88] but was not observed in structure-based Gō models.

In this study, we assumed that the melting temperature of the AA model was equivalent to the experimental melting temperature, because of the difficulty in effective sampling for AA models of large proteins. This was used to normalize the temperature scales for the various coarse-grained models to their corresponding melting temperatures. We have found that the unfolding mechanism of the AA model is not particularly sensitive to variations in temperature of ±10K. In the future however, it would be worthwhile to attempt to surmount this difficulty using a combination of biased sampling techniques and non-equilibrium relations to reconstruct the free energy landscape [89, 90]

An interesting future study will be to apply the tools developed here to full length SOD1, which includes a long loop of 35 amino acids between *β*-strands 4 and 5, and another long loop of 22 amino acids between *β*-strands 7 and 8.

There is nothing necessarily absolute about the force-induced unfolding mechanism found here, which may differ from the unfolding mechanism in either thermal or chemical denaturation. Even within the context of force-induced unfolding, the mechanism may be linkage dependent [91, 92], and may depend on the magnitude of the applied force [41, 93].

## Supporting Information

S1 Fig Comparison of native contacts of the CG models with the AA model. Panels a) and c) compare contact maps of the AA (black open circle) model with the AWSEM (red) (a) and *C*_*α*_-Gō model (cyan) (c) respectively. Panel b) and d) are scatter plots of number of contacts for a pulling trajectory for AA and AWSEM (b), and AA and *C*_*α*_-Gō (d) with (black) and without (red) weighting factors.
(TIF)

S2 Fig Melting curves. for a) HA-Gō, b) AWSEM, and c) *C*_*α*_-Gō model. For each model, 〈*Q*〉 calculated for first (pink circle), second (blue square) and last third (black circles) of the simulation time is shown. The solid black line shows a fitted curve to Eq 5 on the data over the transition region. To obtain the melting temperatures of the CG models, we ran replica-exchange molecular dynamics (REMD) simulations on the HA-Gō and *C*_*α*_-Gō models. For the *C*_*α*_-Gō REMD simulations, the time for the preproduction run was 5 ns for each replica and the production runs for each replica was 5 ns for 16 replicas with replicas over the temperature range of 98–178 K. HA-Gō REMD simulations were performed with 22 replicas, in the temperature range of 70–131 K, with a total simulation time of 315 ns. To calculate 〈*Q*(*T*)〉 for the AWSEM model, we ran 50 direct MD simulations at each temperature *T* over a temperature range of 280–440 K.
(TIF)

S3 Fig Force extension curve and *Q* versus distance at different Temperature for the AA model. In order to test the sensitivity of our results, we have performed 5 simulations of the AA model at T = 290K, 5 simulations at T = 310 K and compare the results of the forced unfolding with pulling at T = 300K (corresponding to *T*_*f*_ = 335 K). The overall behaviour of the force-extension curves and *Q* vs. extension curves are similar. Generally speaking, all force extension curves at different temperatures exhibit the same main features, e.g. main peaks at about 10 and 20 nm. The *Q*-extension curves follow the same pattern approximately. At T = 290K, the force peaks are slightly higher in comparison to the force peaks at higher temperatures. Also, at the higher temperature, the *Q* vs distance curves drop more smoothly. Note that since mechanical unfolding is a stochastic process, we do not expect to see identical curves for all the runs.
(TIFF)

S4 Fig Sequence of unfolding for the AA model at T = 290, 300, and 310 K. Unfolding pathways and sequence of unfolding for lower and upper temperatures are similar to those at T = 300 K. The correlation coefficient between the values in the plots at T = 300 & T = 290 K is 0.96; the correlation coefficient between the values in the plots at T = 300 & 310 K is also 0.96. The color scheme is the same as in Fig 7A.
(TIF)

S5 Fig Fraction of native contacts for each residue vs. total *Q* for the AA model at T = 290, 300, and 310 K. Sequences of unfolding for lower and upper temperatures are similar to those at T = 300 K. Correlation coefficients for T = 300 & 290 K and for T = 300 & 310 K are 0.97 and 0.95. Thus, we are confident that our results for the AA-model are robust with respect to small variations in temperature. The color scheme is the same as in Fig 7B.
(TIFF)

S6 Fig Relaxation rate vs degree of unfolding *Q*. Characteristic relaxation rates are estimated for partially unfolded structures, by selecting five pairs of residues and implementing the same protocol for perturbation and equilibration as described in the main text for the native state. Mean relaxation rates are plotted for all models at *Q* = 1, 0.7, 0.5, 0.3. For the AWSEM model, the remaining region of the protein that is folded is distinct from the other models at low *Q*. The selected pairs of residues for the AWSEM model (at all *Q*) are 4 & 32, 6 & 23, 20 & 29, and 18 & 30. For all other models, the selected pairs of residues are 32 & 44, 35 & 69, 41 & 58, 52 & 74, and 61 & 75. For all models, pairs of residues are chosen from the largest folded segment of the structures at *Q* = 0.3.
(TIF)

S7 Fig Convergence of the force scaling factor *α* as a function of the number of pulling runs obtained from Eq 7 for the HA-Gō (a), AWSEM (b), and *C*_*α*_-Gō (c) model respectively. (TIF)

S1 Table Set of fit parameters *A*_1_/*A*_2_ (see Eq 5) for each perturbed residue pair. (TIF)

S2 Table Set of fit parameters *κ*_1_/*κ*_2_ (see Eq 5) for each perturbed residue pair. (TIF)

## References

[1] Gō N. Theoretical studies of protein folding. Annual Review of Biophysics and Bioengineering. 1983;12(1):183–210. 10.1146/annurev.bb.12.060183.001151 6347038

[2] Clementi C, Nymeyer H, Onuchic JN. Topological and energetic factors: what determines the structural details of the transition state ensemble and “en-route” intermediates for protein folding? An investigation for small globular proteins. Journal of Molecular Biology. 2000;298(5):937–953. 10.1006/jmbi.2000.3693 10801360

[3] Bryngelson JD, Onuchic JN, Socci ND, Wolynes PG. Funnels, pathways, and the energy landscape of protein folding: a synthesis. Proteins: Structure, Function, and Bioinformatics. 1995;21(3):167–195. 10.1002/prot.340210302 7784423

[4] Plotkin SS. Speeding protein folding beyond the Gō model: How a little frustration sometimes helps. Proteins: Structure, Function, and Bioinformatics. 2001;45(4):337–345. 11746681 10.1002/prot.1154

[5] Clementi C. Coarse-grained models of protein folding: toy models or predictive tools? Current Opinion in Structural Biology. 2008;18(1):10–15. 10.1016/j.sbi.2007.10.005 18160277

[6] Mirny L, Shakhnovich E. Protein folding theory: from lattice to all-atom models. Annual Review of Biophysics and Biomolecular Structure. 2001;30(1):361–396. 10.1146/annurev.biophys.30.1.361 11340064

[7] Zhang J, Li W, Wang J, Qin M, Wu L, Yan Z, et al Protein folding simulations: From coarse-grained model to all-atom model. IUBMB life. 2009;61(6):627–643. 10.1002/iub.223 19472192

[8] Naganathan AN. Coarse-grained models of protein folding as detailed tools to connect with experiments. Wiley Interdisciplinary Reviews: Computational Molecular Science. 2013;3(5):504–514. 10.1002/wcms.1133

[9] Zheng W, Glenn P. Probing the folded state and mechanical unfolding pathways of T4 lysozyme using all-atom and coarse-grained molecular simulation. The Journal of Chemical Physics. 2015;142(3):035101 10.1063/1.4905606 25612731

[10] Kouza M, Hu CK, Li MS, Kolinski A. A structure-based model fails to probe the mechanical unfolding pathways of the titin I27 domain. The Journal of Chemical Physics. 2013;139(6):065103 10.1063/1.4817773 23947893

[11] Cieplak M, Hoang TX, Robbins MO. Thermal folding and mechanical unfolding pathways of protein secondary structures. Proteins: Structure, Function, and Bioinformatics. 2002;49(1):104–113. 10.1002/prot.10188 12211020

[12] Piana S, Lindorff-Larsen K, Shaw DE. Atomic-level description of ubiquitin folding. Proceedings of the National Academy of Sciences. 2013;110(15):5915–5920. 10.1073/pnas.1218321110 23503848 PMC3625349

[13] Lindorff-Larsen K, Piana S, Dror RO, Shaw DE. How fast-folding proteins fold. Science. 2011;334(6055):517–520. 10.1126/science.1208351 22034434

[14] Lindorff-Larsen K, Maragakis P, Piana S, Eastwood MP, Dror RO, Shaw DE. Systematic validation of protein force fields against experimental data. PloS one. 2012;7(2):e32131 10.1371/journal.pone.0032131 22384157 PMC3285199

[15] Pan AC, Borhani DW, Dror RO, Shaw DE. Molecular determinants of drug–receptor binding kinetics. Drug Discovery Today. 2013;18(13):667–673. 10.1016/j.drudis.2013.02.007 23454741

[16] Bock LV, Blau C, Schröder GF, Davydov II, Fischer N, Stark H, et al Energy barriers and driving forces in tRNA translocation through the ribosome. Nature Structural & Molecular Biology. 2013;20(12):1390–1396. 10.1038/nsmb.2690 24186064

[17] Žoldák G, Rief M. Force as a single molecule probe of multidimensional protein energy landscapes. Current Opinion in Structural Biology. 2013;23(1):48–57. 10.1016/j.sbi.2012.11.007 23279960

[18] Woodside MT, Block SM. Reconstructing folding energy landscapes by single-molecule force spectroscopy. Annual Review of Biophysics. 2014;43:19 10.1146/annurev-biophys-051013-022754 24895850 PMC4609573

[19] Moffitt JR, Chemla YR, Smith SB, Bustamante C. Recent advances in optical tweezers. Biochemistry. 2008;77(1):205 10.1146/annurev.biochem.77.043007.090225 18307407

[20] Lu H, Isralewitz B, Krammer A, Vogel V, Schulten K. Unfolding of titin immunoglobulin domains by steered molecular dynamics simulation. Biophysical Journal. 1998;75(2):662–671. 10.1016/S0006-3495(98)77556-3 9675168 PMC1299741

[21] Ritchie DB, Woodside MT. Probing the structural dynamics of proteins and nucleic acids with optical tweezers. Current Opinion in Structural Biology. 2015;34:43–51. 10.1016/j.sbi.2015.06.006 26189090 PMC7126019

[22] Izrailev S, Stepaniants S, Balsera M, Oono Y, Schulten K. Molecular dynamics study of unbinding of the avidin-biotin complex. Biophysical Journal. 1997;72(4):1568 10.1016/S0006-3495(97)78804-0 9083662 PMC1184352

[23] Shaw DE, Deneroff MM, Dror RO, Kuskin JS, Larson RH, Salmon JK, et al Anton, a special-purpose machine for molecular dynamics simulation. Communications of the ACM. 2008;51(7):91–97.

[24] Clementi C, Nymeyer H, Onuchic JN. Topological and energetic factors: what determines the structural details of the transition state ensemble and “en-route” intermediates for protein folding? an investigation for small globular proteins1. Journal of Molecular Biology. 2000;298(5):937–953. 10.1006/jmbi.2000.3693 10801360

[25] Whitford PC, Noel JK, Gosavi S, Schug A, Sanbonmatsu KY, Onuchic JN. An all-atom structure-based potential for proteins: Bridging minimal models with all-atom empirical forcefields. Proteins: Structure, Function, and Bioinformatics. 2009;75(2):430–441. 10.1002/prot.22253 18837035 PMC3439813

[26] Eastwood MP, Wolynes PG. Role of explicitly cooperative interactions in protein folding funnels: A simulation study. Journal of Chemical Physics. 2001;114(10):4702–4716. 10.1063/1.1315994

[27] Davtyan A, Schafer NP, Zheng W, Clementi C, Wolynes PG, Papoian GA. AWSEM-MD: Protein Structure Prediction Using Coarse-Grained Physical Potentials and Bioinformatically Based Local Structure Biasing. The Journal of Physical Chemistry B. 2012;116(29):8494–8503. 10.1021/jp212541y 22545654 PMC3406225

[28] Paci E, Vendruscolo M, Karplus M. Native and non-native interactions along protein folding and unfolding pathways. Proteins: Structure, Function, and Bioinformatics. 2002;47(3):379–392. 10.1002/prot.10089 11948791

[29] Forman JR, Qamar S, Sandford RN, Paci E, Clarke J, et al Non-native interactions are critical for mechanical strength in PKD domains. Structure. 2009;17(12):1582–1590. 10.1016/j.str.2009.09.013 20004162 PMC2862302

[30] Fowler SB, Best RB, Herrera JLT, Rutherford TJ, Steward A, Paci E, et al Mechanical unfolding of a titin Ig domain: structure of unfolding intermediate revealed by combining AFM, molecular dynamics simulations, NMR and protein engineering. Journal of Molecular Biology. 2002;322(4):841–849. 10.1016/S0022-2836(02)00805-7 12270718

[31] Sun L, Noel JK, Sulkowska JI, Levine H, Onuchic JN. Connecting thermal and mechanical protein (un) folding landscapes. Biophysical Journal. 2014;107(12):2950–2961. 10.1016/j.bpj.2014.10.021 25517160 PMC4269773

[32] Rosen DR, Siddique T, Patterson D, Figlewicz DA, Sapp P, Hentati A, et al Mutations in Cu/Zn superoxide dismutase gene are associated with familial Amyotrophic Lateral Sclerosis. Nature. 1993;362(6415):59–62. 10.1038/362059a0 8446170

[33] Deng HX, Hentati A, Tainer JA, Iqbal Z, Cayabyab A, Hung WY, et al Amyotrophic Lateral Sclerosis and structural defects in Cu, Zn superoxide dismutase. Science. 1993;261(5124):1047–1051. 10.1126/science.8351519 8351519

[34] Cleveland DW, Rothstein JD. From Charcot to Lou Gehrig: deciphering selective motor neuron death in ALS. Nature Reviews Neuroscience. 2001;2(11):806–819. 10.1038/35097565 11715057

[35] Rowland LP, Shneider NA. Amyotrophic Lateral Sclerosis. New England Journal of Medicine. 2001;344(22):1688–1700. 10.1056/NEJM200105313442207 11386269

[36] Danielsson J, Kurnik M, Lang L, Oliveberg M. Cutting off functional loops from homodimeric enzyme superoxide dismutase 1 (SOD1) leaves monomeric *β*-barrels. Journal of Biological Chemistry. 2011;286(38):33070–33083. 10.1074/jbc.M111.251223 21700707 PMC3190884

[37] Danielsson J, Awad W, Saraboji K, Kurnik M, Lang L, Leinartaitė L, et al Global structural motions from the strain of a single hydrogen bond. Proceedings of the National Academy of Sciences. 2013;110(10):3829–3834. 10.1073/pnas.1217306110 23431167 PMC3593908

[38] Eghiaian F, Rico F, Colom A, Casuso I, Scheuring S. High-speed atomic force microscopy: Imaging and force spectroscopy. FEBS letters. 2014;588(19):3631–3638. 10.1016/j.febslet.2014.06.028 24937145

[39] Sotomayor M, Schulten K. Single-molecule experiments in vitro and in silico. Science. 2007;316(5828):1144–1148. 10.1126/science.1137591 17525328

[40] Dudko OK, Hummer G, Szabo A. Theory, analysis, and interpretation of single-molecule force spectroscopy experiments. Proceedings of the National Academy of Sciences. 2008;105(41):15755–15760. 10.1073/pnas.0806085105 18852468 PMC2572921

[41] Irbäck A, Mitternacht S, Mohanty S. Dissecting the mechanical unfolding of ubiquitin. Proceedings of the National Academy of Sciences of the United States of America. 2005;102(38):13427–13432. 10.1073/pnas.0501581102 16174739 PMC1224613

[42] Pettersen EF, Goddard TD, Huang CC, Couch GS, Greenblatt DM, Meng EC, et al UCSF Chimera—a visualization system for exploratory research and analysis. Journal of Computational Chemistry. 2004;25(13):1605–1612. 10.1002/jcc.20084 15264254

[43] Piana S, Lindorff-Larsen K, Shaw DE. How Robust Are Protein Folding Simulations with Respect to Force Field Parameterization? Biophysical Journal. 2011;100(9):L47–L49. 10.1016/j.bpj.2011.03.051 21539772 PMC3149239

[44] Neria E, Fischer S, Karplus M. Simulation of activation free energies in molecular systems. The Journal of Chemical Physics. 1996;105(5):1902–1921. 10.1063/1.472061

[45] Jorgensen WL, Chandrasekhar J, Madura JD, Impey RW, Klein ML. Comparison of simple potential functions for simulating liquid water. The Journal of Chemical Physics. 1983;79(2):926–935. 10.1063/1.445869

[46] Pronk S, Páll S, Schulz R, Larsson P, Bjelkmar P, Apostolov R, et al GROMACS 4.5: a high-throughput and highly parallel open source molecular simulation toolkit. Bioinformatics. 2013;29(7):845–854. 10.1093/bioinformatics/btt055 23407358 PMC3605599

[47] van der Spoel D, Lindahl E, Hess B. GROMACS User Manual version 4.6. 7; 2014.

[48] Hess B, Bekker H, Berendsen HJ, Fraaije JG, et al LINCS: a linear constraint solver for molecular simulations. Journal of Computational Chemistry. 1997;18(12):1463–1472. 10.1002/(SICI)1096-987X(199709)18:12%3C1463::AID-JCC4%3E3.3.CO;2-L

[49] Cheng A, Merz KM. Application of the Nose-Hoover chain algorithm to the study of protein dynamics. The Journal of Physical Chemistry. 1996;100(5):1927–1937. 10.1021/jp951968y

[50] Lingenheil M, Denschlag R, Reichold R, Tavan P. The “hot-solvent/cold-solute” problem revisited. Journal of Chemical Theory and Computation. 2008;4(8):1293–1306. 10.1021/ct8000365 26631705

[51] Mor A, Ziv G, Levy Y. Simulations of proteins with inhomogeneous degrees of freedom: the effect of thermostats. Journal of Computational Chemistry. 2008;29(12):1992–1998. 10.1002/jcc.20951 18366022

[52] Bussi G, Donadio D, Parrinello M. Canonical sampling through velocity rescaling. The Journal of Chemical Physics. 2007;126(1). 10.1063/1.2408420 17212484

[53] Parrinello M, Rahman A. Polymorphic transitions in single crystals: A new molecular dynamics method. Journal of Applied physics. 1981;52(12):7182–7190. 10.1063/1.328693

[54] Essmann U, Perera L, Berkowitz ML, Darden T, Lee H, Pedersen LG. A smooth particle mesh Ewald method. The Journal of Chemical Physics. 1995;103(19):8577–8593. 10.1063/1.470117

[55] Noel JK, Onuchic JN. The many faces of structure-based potentials: from protein folding landscapes to structural characterization of complex biomolecules In: Computational Modeling of Biological Systems. Springer; 2012 p. 31–54. 10.1007/978-1-4614-2146-7_2

[56] Noel JK, Whitford PC, Sanbonmatsu KY, Onuchic JN. SMOG@ctbp: simplified deployment of structure-based models in GROMACS. Nucleic Acids Research. 2010;38:657–661. 10.1093/nar/gkq498 20525782 PMC2896113

[57] Plimpton S. Fast Parallel Algorithms for Short-Range Molecular Dynamics. Journal of Computational Physics. 1995;117(1):1–19. 10.1006/jcph.1995.1039

[58] Best RB, Hummer G, Eaton WA. Native contacts determine protein folding mechanisms in atomistic simulations. Proceedings of the National Academy of Sciences. 2013;110(44):17874–17879. 10.1073/pnas.1311599110 24128758 PMC3816414

[59] Flyvbjerg H, Petersen HG. Error estimates on averages of correlated data. The Journal of Chemical Physics. 1989;91(1):461–466. 10.1063/1.457480

[60] Mills EA, Plotkin SS. Protein Transfer Free Energy Obeys Entropy-enthalpy Compensation. The Journal of Physical Chemistry B. 2015;119(44):14130–14144. 10.1021/acs.jpcb.5b09219 26423005

[61] Danielsson J, Mu X, Lang L, Wang H, Binolfi A, Theillet FX, et al Thermodynamics of protein destabilization in live cells. Proceedings of the National Academy of Sciences. 2015;112(40):12402–12407. 10.1073/pnas.1511308112 26392565 PMC4603463

[62] Frank MK, Dyda F, Dobrodumov A, Gronenborn AM. Core mutations switch monomeric protein GB1 into an intertwined tetramer. Nature Structural & Molecular Biology. 2002;9(11):877–885. 10.1038/nsb854 12379842

[63] Jarzynski C. Nonequilibrium equality for free energy differences. Physical Review Letters. 1997;78(14):2690 10.1103/PhysRevLett.78.2690

[64] Gore J, Ritort F, Bustamante C. Bias and error in estimates of equilibrium free-energy differences from nonequilibrium measurements. Proceedings of the National Academy of Sciences. 2003;100(22):12564–12569. 10.1073/pnas.1635159100 14528008 PMC240657

[65] Wei G, Derreumaux P, Mousseau N. Sampling the complex energy landscape of a simple *β*-hairpin. The Journal of Chemical Physics. 2003;119(13):6403–6406. 10.1063/1.1613642

[66] Zhang Y, Skolnick J. Scoring function for automated assessment of protein structure template quality. Proteins: Structure, Function, and Bioinformatics. 2004;57(4):702–710. 10.1002/prot.20264 15476259

[67] Siew N, Elofsson A, Rychlewski L, Fischer D. MaxSub: an automated measure for the assessment of protein structure prediction quality. Bioinformatics. 2000;16(9):776–785. 10.1093/bioinformatics/16.9.776 11108700

[68] Zhang Y, Skolnick J. TM-align: a protein structure alignment algorithm based on the TM-score. Nucleic Acids Research. 2005;33(7):2302–2309. 10.1093/nar/gki524 15849316 PMC1084323

[69] Plotkin SS, Onuchic JN. Investigation of routes and funnels in protein folding by free energy functional methods. Proceedings of the National Academy of Sciences. 2000;97(12):6509–6514. 10.1073/pnas.97.12.6509 10841554 PMC18640

[70] Plotkin SS, Onuchic JN. Understanding protein folding with energy landscape theory part I: basic concepts. Quarterly Reviews of Biophysics. 2002;35(02):111–167. 10.1017/S0033583502003761 12197302

[71] Plotkin SS, Onuchic JN. Understanding protein folding with energy landscape theory Part II: Quantitative aspects. Quarterly Reviews of Biophysics. 2002;35(03):205–286. 10.1017/S0033583502003785 12599750

[72] Chavez LL, Onuchic JN, Clementi C. Quantifying the roughness on the free energy landscape: entropic bottlenecks and protein folding rates. Journal of the American Chemical Society. 2004;126(27):8426–8432. 10.1021/ja049510+ 15237999

[73] Yang WY, Pitera JW, Swope WC, Gruebele M. Heterogeneous folding of the trpzip hairpin: full atom simulation and experiment. Journal of Molecular Biology. 2004;336(1):241–251. 10.1016/j.jmb.2003.11.033 14741219

[74] Suzuki Y, Noel JK, Onuchic JN. An analytical study of the interplay between geometrical and energetic effects in protein folding. The Journal of Chemical Physics. 2008;128(2):025101 10.1063/1.2812956 18205476

[75] Naganathan AN, Muñoz V. Insights into protein folding mechanisms from large scale analysis of mutational effects. Proceedings of the National Academy of Sciences. 2010;107(19):8611–8616. 10.1073/pnas.1000988107 20418505 PMC2889297

[76] Das A, Plotkin SS. SOD1 exhibits allosteric frustration to facilitate metal binding affinity. Proceedings of the National Academy of Sciences. 2013;110(10):3871–3876. 10.1073/pnas.1216597110 23431152 PMC3593857

[77] Rustad M, Ghosh K. Why and how does native topology dictate the folding speed of a protein? The Journal of Chemical Physics. 2012;137(20):205104 10.1063/1.4767567 23206039

[78] Plotkin SS, Onuchic JN. Structural and energetic heterogeneity in protein folding. I. Theory. The Journal of Chemical Physics. 2002;116(12):5263–5283. 10.1063/1.1449866

[79] Clementi C, Plotkin SS. The effects of nonnative interactions on protein folding rates: theory and simulation. Protein Science. 2004;13(7):1750–1766. 10.1110/ps.03580104 15215519 PMC2279923

[80] Di Nardo AA, Korzhnev DM, Stogios PJ, Zarrine-Afsar A, Kay LE, Davidson AR. Dramatic acceleration of protein folding by stabilization of a nonnative backbone conformation. Proceedings of the National Academy of Sciences of the United States of America. 2004;101(21):7954–7959. 10.1073/pnas.0400550101 15148398 PMC419538

[81] Plotkin SS, Wang J, Wolynes PG. Statistical mechanics of a correlated energy landscape model for protein folding funnels. The Journal of Chemical Physics. 1997;106(7):2932–2948. 10.1063/1.473355

[82] Plotkin SS, Wolynes PG. Non-Markovian configurational diffusion and reaction coordinates for protein folding. Physical Review Letters. 1998;80(22):5015 10.1103/PhysRevLett.80.5015

[83] Zagrovic B, Snow CD, Shirts MR, Pande VS. Simulation of folding of a small alpha-helical protein in atomistic detail using worldwide-distributed computing. Journal of Molecular Biology. 2002;323(5):927–937. 10.1016/S0022-2836(02)00997-X 12417204

[84] Kaya H, Chan HS. Solvation effects and driving forces for protein thermodynamic and kinetic cooperativity: how adequate is native-centric topological modeling? Journal of Molecular Biology. 2003;326(3):911–931. 10.1016/S0022-2836(02)01434-1 12581650

[85] Best RB, Hummer G. Coordinate-dependent diffusion in protein folding. Proceedings of the National Academy of Sciences. 2010;107(3):1088–1093. 10.1073/pnas.0910390107 20080558 PMC2824289

[86] Zhang Z, Chan HS. Competition between native topology and nonnative interactions in simple and complex folding kinetics of natural and designed proteins. Proceedings of the National Academy of Sciences. 2010;107(7):2920–2925. 10.1073/pnas.0911844107 20133730 PMC2840274

[87] Kouza M, Hu CK, Zung H, Li MS. Protein mechanical unfolding: Importance of non-native interactions. The Journal of Chemical Physics. 2009;131(21):215103 10.1063/1.3272275 19968370

[88] Schwaiger I, Kardinal A, Schleicher M, Noegel AA, Rief M. A mechanical unfolding intermediate in an actin-crosslinking protein. Nature Structural & Molecular Biology. 2004;11(1):81–85. 10.1038/nsmb705 14718927

[89] Harris NC, Song Y, Kiang CH. Experimental free energy surface reconstruction from single-molecule force spectroscopy using Jarzynski’s equality. Physical Review Letters. 2007;99(6):068101 10.1103/PhysRevLett.99.068101 17930869 PMC2682736

[90] Hummer G, Szabo A. Free energy surfaces from single-molecule force spectroscopy. Accounts of Chemical Research. 2005;38(7):504–513. 10.1021/ar040148d 16028884

[91] Carrion-Vazquez M, Li H, Lu H, Marszalek PE, Oberhauser AF, Fernandez JM. The mechanical stability of ubiquitin is linkage dependent. Nature Structural & Molecular Biology. 2003;10(9):738–743. 10.1038/nsb965 12923571

[92] Brockwell DJ, Paci E, Zinober RC, Beddard GS, Olmsted PD, Smith DA, et al Pulling geometry defines the mechanical resistance of a *β*-sheet protein. Nature Structural & Molecular Biology. 2003;10(9):731–737. 10.1038/nsb968 12923573

[93] Hyeon C, Thirumalai D. Mechanical unfolding of RNA: from hairpins to structures with internal multiloops. Biophysical Journal. 2007;92(3):731–743. 10.1529/biophysj.106.093062 17028142 PMC1779982

