# Supplementary figures and images for: As Simple As Possible, but Not Simpler: Exploring the Fidelity of Coarse-Grained Protein Models for Simulated Force Spectroscopy
Source: PLoS Comput Biol. 2016 Nov 29;12(11):e1005211. doi: 10.1371/journal.pcbi.1005211 (PMC5127490; doi:10.1371/journal.pcbi.1005211)

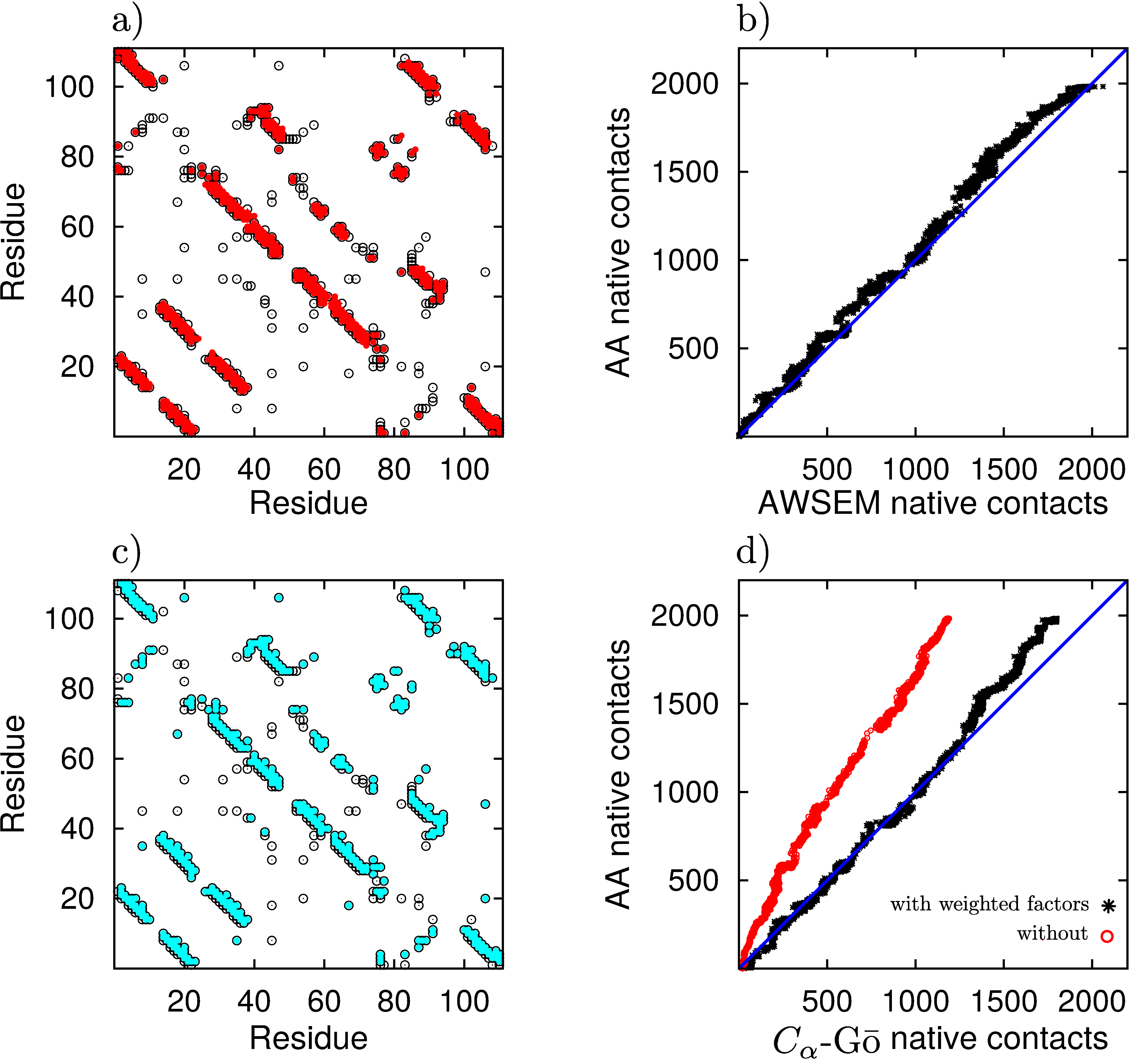

Supplement: S1 Fig — Panels a) and c) compare contact maps of the AA (black open circle) model with the AWSEM (red) (a) and Cα-Gō model (cyan) (c) respectively. Panel b) and d) are scatter plots of number of contacts for a pulling trajectory for AA and AWSEM (b), and AA and Cα-Gō (d) with (black) and without (red) weighting factors. (TIF) [file pcbi.1005211.s001.tif]

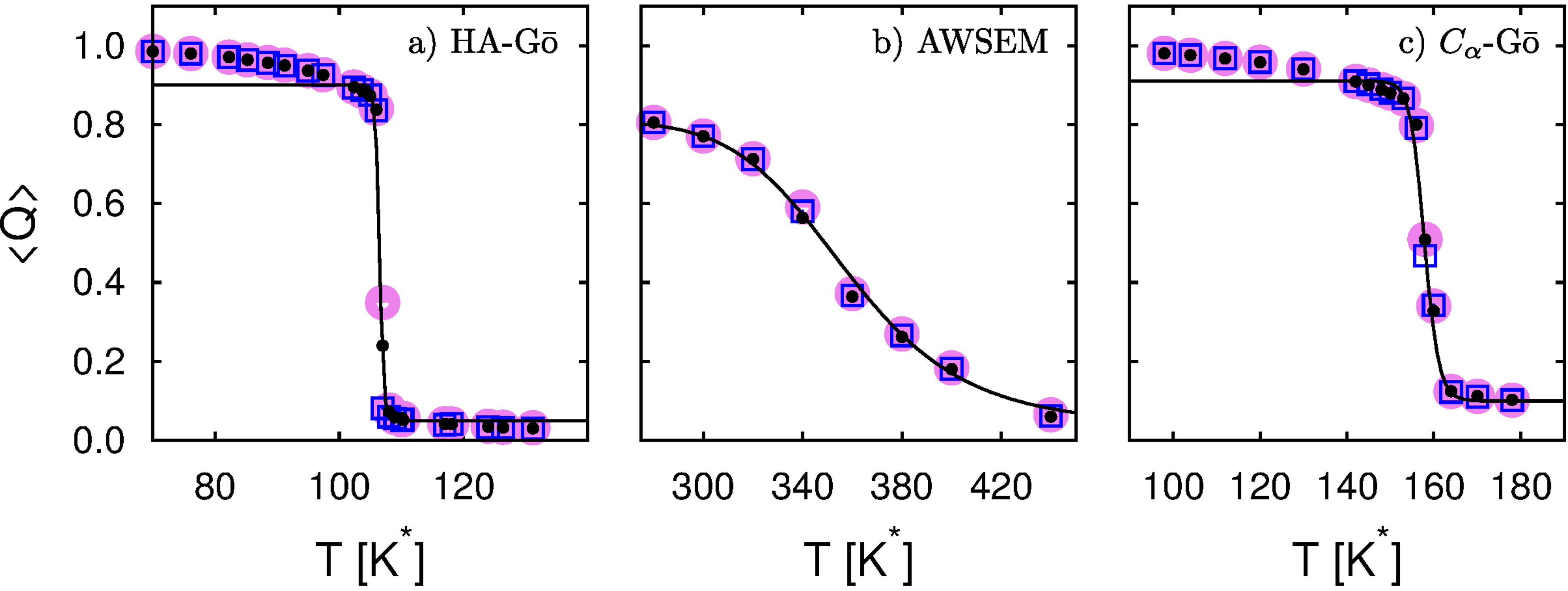

Supplement: S2 Fig — for a) HA-Gō, b) AWSEM, and c) Cα-Gō model. For each model, 〈Q〉 calculated for first (pink circle), second (blue square) and last third (black circles) of the simulation time is shown. The solid black line shows a fitted curve to Eq 5 on the data over the transition region. To obtain the melting temperatures of the CG models, we ran replica-exchange molecular dynamics (REMD) simulations on the HA-Gō and Cα-Gō models. For the Cα-Gō REMD simulations, the time for the preproduction run was 5 ns for each replica and the production runs for each replica was 5 ns for 16 replicas with replicas over the temperature range of 98–178 K. HA-Gō REMD simulations were performed with 22 replicas, in the temperature range of 70–131 K, with a total simulation time of 315 ns. To calculate 〈Q(T)〉 for the AWSEM model, we ran 50 direct MD simulations at each temperature T over a temperature range of 280–440 K. (TIF) [file pcbi.1005211.s002.tif]

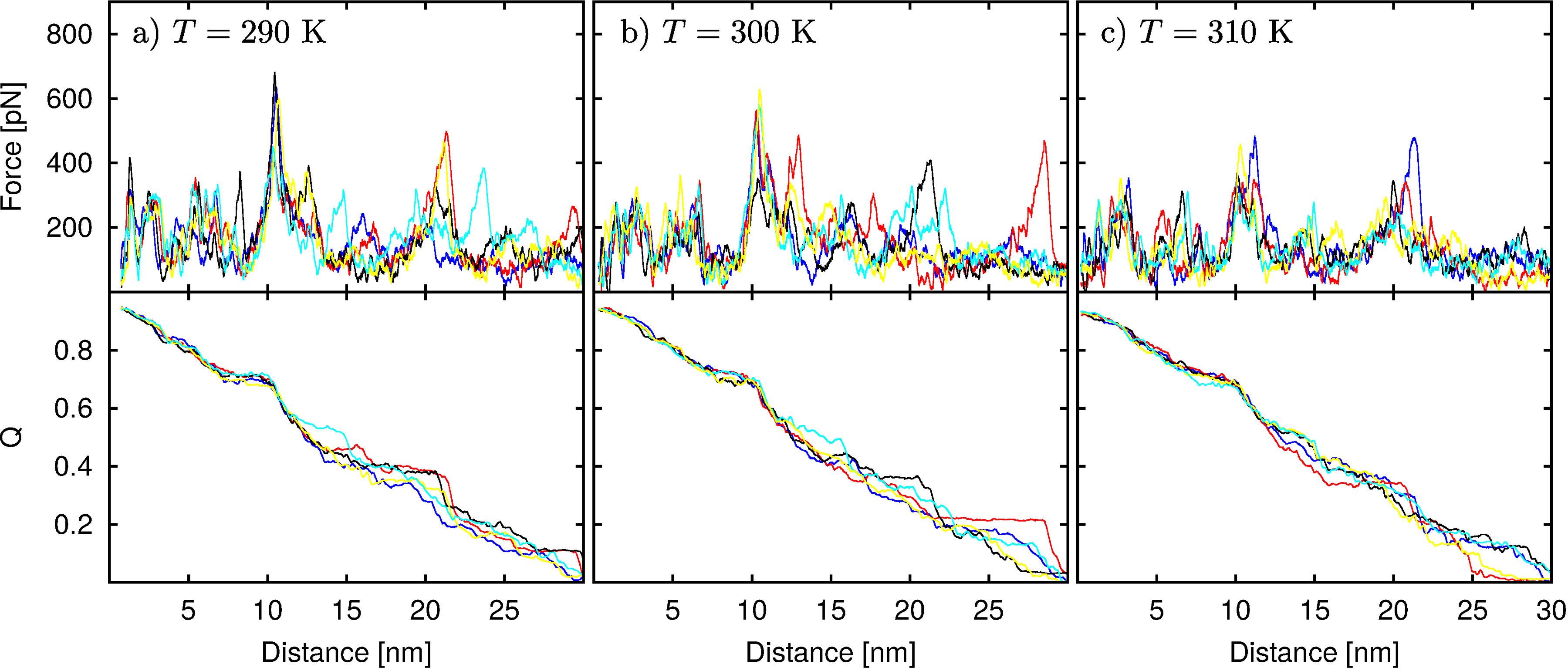

Supplement: S3 Fig — In order to test the sensitivity of our results, we have performed 5 simulations of the AA model at T = 290K, 5 simulations at T = 310 K and compare the results of the forced unfolding with pulling at T = 300K (corresponding to Tf = 335 K). The overall behaviour of the force-extension curves and Q vs. extension curves are similar. Generally speaking, all force extension curves at different temperatures exhibit the same main features, e.g. main peaks at about 10 and 20 nm. The Q-extension curves follow the same pattern approximately. At T = 290K, the force peaks are slightly higher in comparison to the force peaks at higher temperatures. Also, at the higher temperature, the Q vs distance curves drop more smoothly. Note that since mechanical unfolding is a stochastic process, we do not expect to see identical curves for all the runs. (TIFF) [file pcbi.1005211.s003.tiff]

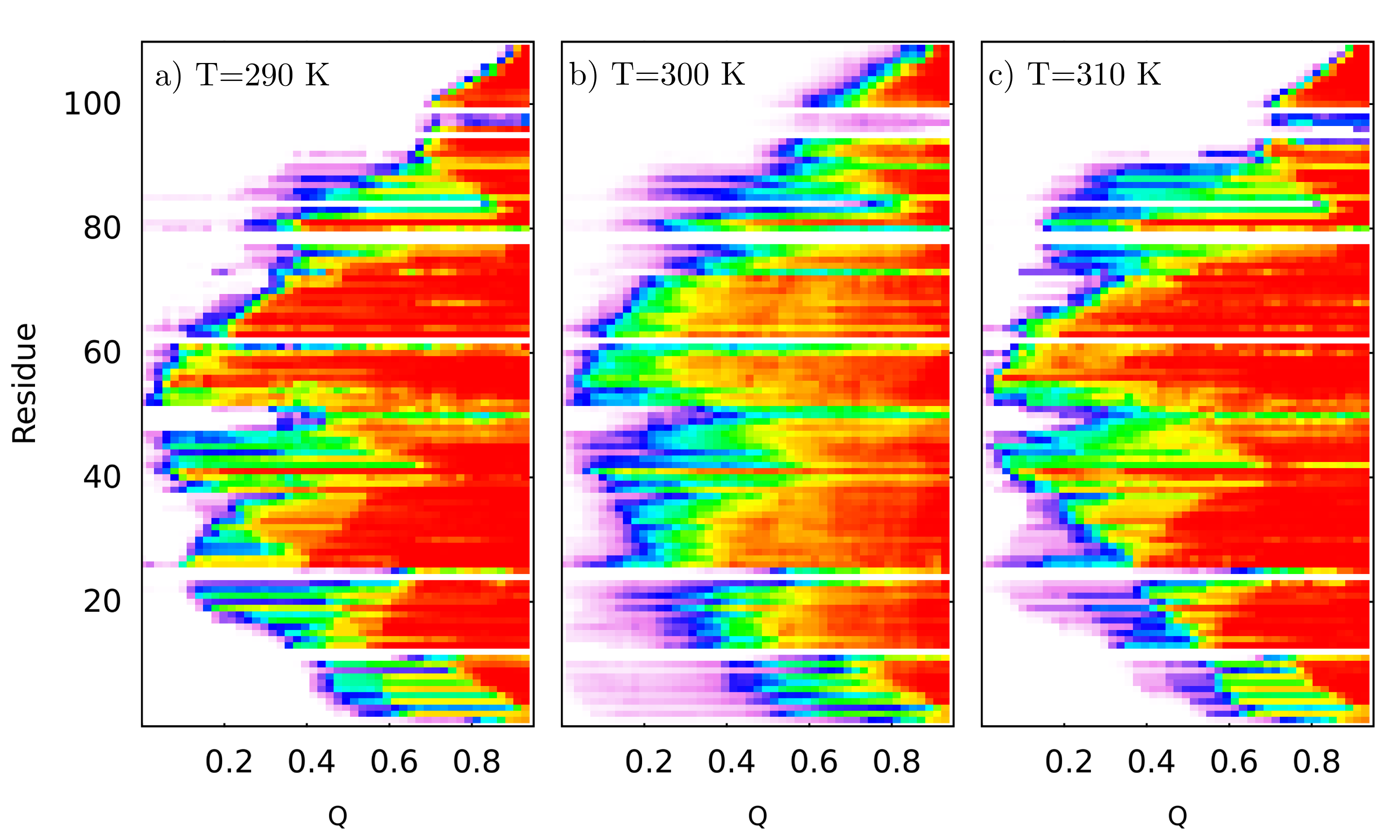

Supplement: S4 Fig — Unfolding pathways and sequence of unfolding for lower and upper temperatures are similar to those at T = 300 K. The correlation coefficient between the values in the plots at T = 300 & T = 290 K is 0.96; the correlation coefficient between the values in the plots at T = 300 & 310 K is also 0.96. The color scheme is the same as in Fig 7A. (TIF) [file pcbi.1005211.s004.tif]

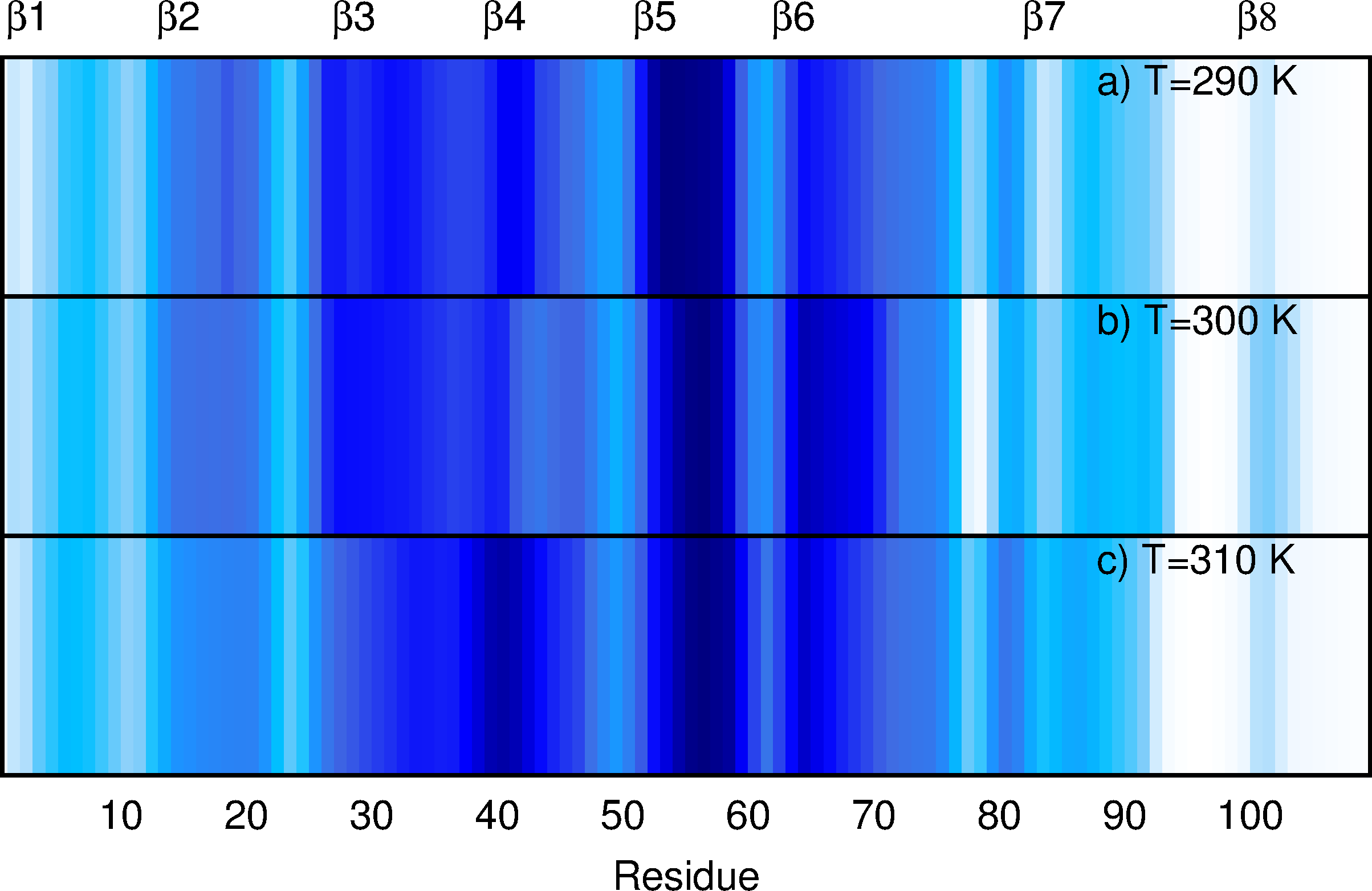

Supplement: S5 Fig — Sequences of unfolding for lower and upper temperatures are similar to those at T = 300 K. Correlation coefficients for T = 300 & 290 K and for T = 300 & 310 K are 0.97 and 0.95. Thus, we are confident that our results for the AA-model are robust with respect to small variations in temperature. The color scheme is the same as in Fig 7B. (TIFF) [file pcbi.1005211.s005.tiff]

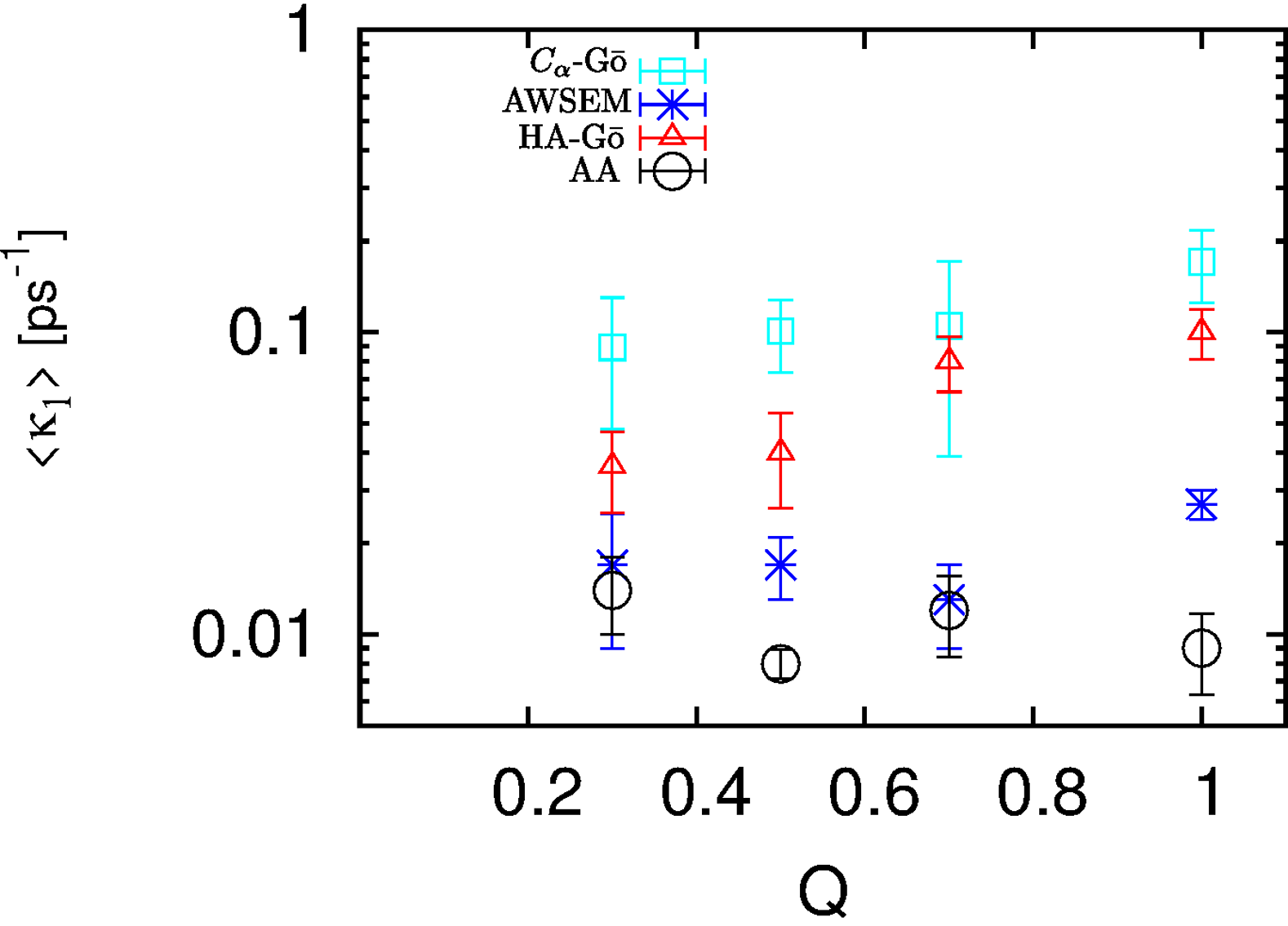

Supplement: S6 Fig — Characteristic relaxation rates are estimated for partially unfolded structures, by selecting five pairs of residues and implementing the same protocol for perturbation and equilibration as described in the main text for the native state. Mean relaxation rates are plotted for all models at Q = 1, 0.7, 0.5, 0.3. For the AWSEM model, the remaining region of the protein that is folded is distinct from the other models at low Q. The selected pairs of residues for the AWSEM model (at all Q) are 4 & 32, 6 & 23, 20 & 29, and 18 & 30. For all other models, the selected pairs of residues are 32 & 44, 35 & 69, 41 & 58, 52 & 74, and 61 & 75. For all models, pairs of residues are chosen from the largest folded segment of the structures at Q = 0.3. (TIF) [file pcbi.1005211.s006.tif]

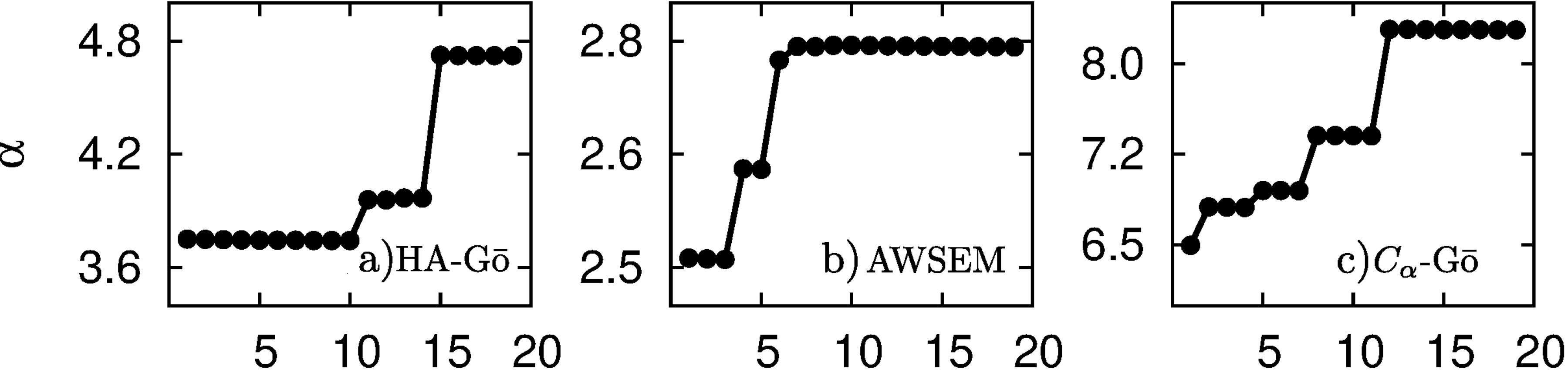

Supplement: S7 Fig — (TIF) [file pcbi.1005211.s007.tif]

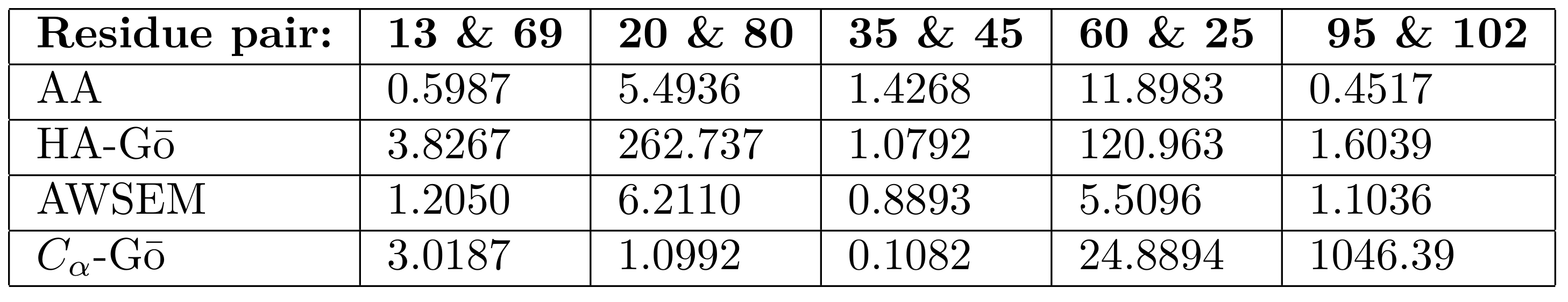

Supplement: S1 Table — (TIF) [file pcbi.1005211.s008.tif]

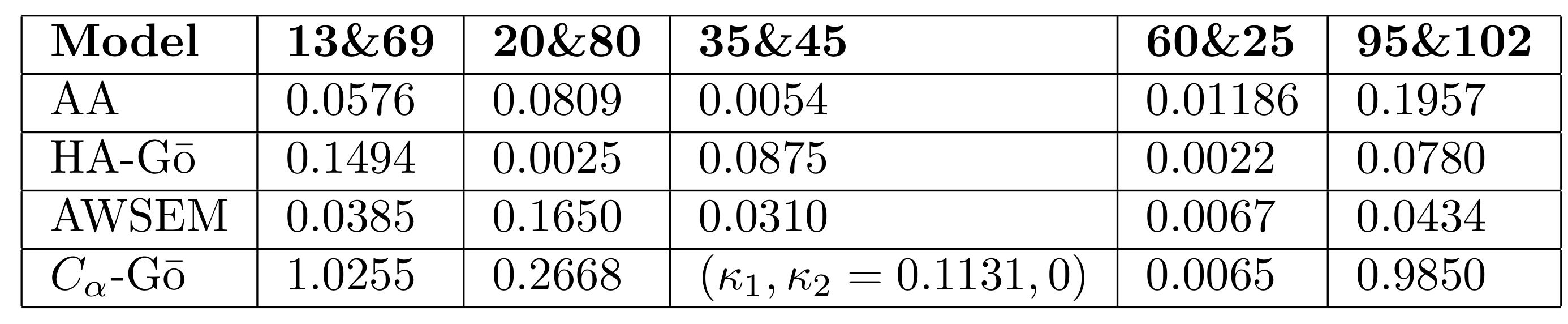

Supplement: S2 Table — (TIF) [file pcbi.1005211.s009.tif]
